# Hydrogel-Based Active Substance Release Systems for Cosmetology and Dermatology Application: A Review

**DOI:** 10.3390/pharmaceutics12050396

**Published:** 2020-04-26

**Authors:** Martyna Zagórska-Dziok, Marcin Sobczak

**Affiliations:** 1 Department of Cosmetics and Pharmaceutical Products Technology, Medical College, University of Information Technology and Management in Rzeszow, 2 Sucharskiego St., 35-225 Rzeszów, Poland; 2 Chair of Analytical Chemistry and Biomaterials, Department of Biomaterials Chemistry, Faculty of Pharmacy, Medical University of Warsaw, 1 Banacha St., 02-097 Warsaw, Poland

**Keywords:** biomedical materials, hydrogels, hydrogels for cosmetology, hydrogels for dermatology, controlled release of active substances, transdermal therapeutic systems

## Abstract

Hydrogels are playing an increasingly important role in medicine and pharmacy. Due to their favorable physicochemical properties, biocompatibility, and designed interaction with living surroundings, they seem to be one of the most promising groups of biomaterials. Hydrogel formulations from natural, semi, or synthetic polymeric materials have gained great attention in recent years for treating various dermatology maladies and for cosmetology procedures. The purpose of this review is to present a brief review on the basic concept of hydrogels, synthesis methods, relevant mechanisms, and applications in dermatology or cosmetology. This review discusses transdermal therapies and the recent advances that have occurred in the field.

## 1. Introduction

Dermatology and cosmetology are dynamically developing fields that deal with the diagnosis and treatment of skin, nail, and hair diseases. Dermatology is a medical discipline dealing with the diseases of the above-mentioned parts of the body, and with the treatment of certain systemic diseases, especially those whose symptoms can be observed primarily on the skin. In turn, cosmetology focuses mainly on skin, hair, and nail care in various disease states. Treatments carried out by both are aimed at improving the external appearance of the skin by treating its various diseases and pathological conditions. Nowadays, attention to external appearances is very noticeable. Many people put a lot of effort into ensuring that their skin is in the best condition. Although reports indicate that skin disease rates are lower compared to other diseases, the undeniable fact is that all skin conditions have a significant impact on quality of life. Moreover, it should be noted that some of them, especially skin cancers or serious infections, can pose a significant threat not only to health, but also to life [1]. Therefore, there is a need for new and innovative products that will be effective remedies for skin diseases.

A few decades ago, cosmetics were mainly applied to the surface of the skin to ensure its vitality or younger appearance. Their importance has increased significantly and currently they also play an important role in dermatology, supporting the fight against various skin disorders [2]. The increase in the importance of cosmetics in skin care is mainly due to scientific and technological progress, thanks to which we observe a better understanding of skin physiology and are able to notice the significant impact of cosmetic preparations on the modification of its physical and biological properties. The wide spectrum of available research methods makes it possible to assess the skin’s response to many stimuli, which have a significant and noticeable impact on the development of cosmetics and dermocosmetics in the market. The currently designed preparations are based on research aimed at a deep understanding of the physiology of skin and its reactions to various stimuli, both internal and external. In vitro studies on cell lines and in vivo studies using animal models are very useful in assessing the impact of newly designed preparations. There are also more and more studies involving people, thanks to which we can get information on the impact of individual cosmetics or dermocosmetics on the condition of our skin.

Skin diseases are one of the most common disorders affecting people around the world. Although the mortality rates are lower than for many other diseases, skin problems have a significant impact on the quality of life of people who suffer from them. Very often, these diseases are associated with many skin lesions, persistent itching, and pain, which significantly reduce quality of life, stimulate social isolation, and are comparable to the feelings of people suffering from various chronic non-dermatological diseases [3]. The prevalence of skin and skin-related disorders may vary depending on age and gender as well as geographical areas [4]. Skin conditions affect people of all ages living in both developed and developing countries. Therefore, there is an urgent need to develop effective remedies and treatment methods to minimize the effects of these diseases. This is extremely important, because as the data show, the percentage of treatment failures is still very high [3]. Analyses regarding global disability and mortality caused by skin diseases carried out by team members of the Institute of Health Metrics and international skin experts in dermatoepidemiology showed that the most common skin conditions are various dermatitis conditions mainly atopic, psoriasis, seborrheic and contact, pyoderma, or cellulitis. A high incidence of such diseases as scabies, viral and fungal skin disorders, decubitus ulcer, urticaria, acne vulgaris, alopecia areata, and pruritus is also observed. Among the more serious skin conditions that are common in the population are malignant skin melanoma and keratinocyte carcinoma, both basal and squamous cell carcinomas [5].

Hydrogels are one of the most interesting groups of medical materials for cosmetology and dermatology. One of the main advantages of hydrogels used in the topical treatment of skin diseases is their ease of application and significant minimization of side effects. With oral or intravenous use of various medicinal substances, their serum concentrations often reach high values, which increase the risk of significant complications. The application of therapeutic compounds incorporated into the hydrogel structure directly on the skin also protects these compounds against the action of liver enzymes and the first-pass effect in the liver. During transdermal administration, the drug initially penetrates the stratum corneum, then the deeper epidermis until it reaches the dermis. After reaching the dermal layer, it can be absorbed into the systemic circulation by dermal microcirculation [6]. It should be noted, however, that local drug delivery depends on many factors, including skin barrier properties as well as physicochemical properties of the embedded therapeutic compound and its carrier. Due to the high water content of hydrogel structures, these biomaterials were mainly seen as carriers of hydrophilic drugs. However, due to the multitude of hydrophobic drugs used in the treatment of a wide spectrum of diseases, research is currently underway on the development of new matrices capable of incorporating these types of therapeutic substances [7]. Due to the fact that hydrophobic compounds have limited loading quantity and homogeneity in hydrogel matrices, the compatibility of hydrogels with hydrophobic drugs should be improved [8,9]. This is possible, among other ways, by introducing molecules capable of forming inclusion complexes or by incorporating hydrophobic moieties into hydrogel structures. Other possibilities are the use of hydrogels containing micelles or nanoparticles in their structures [8,10]. Another option is to combine the particles with a hydrogel and trap the liposomes, nanoparticles, or microspheres [11]. A way to create a hydrogel carrier for hydrophobic drugs is also to build a mixed micellar gel consisting of a polymer and a surfactant [12]. The interactions between the analyzed drug embedded in the carrier structure and the skin surface and its individual layers are also extremely important. The molecular weight of the drug and its lipophilicity or hydrophilicity also plays an important role in the effectiveness of the topical application of active substances. This type of drug application results in achieving therapeutic concentration in individual layers of the skin located in the area of application, while it significantly reduces the serum concentration of the drug, which minimizes undesirable effects. However, it should be remembered that the topical application of therapeutic compounds is often associated with various types of skin irritation, but these are usually mild lesions [13,14].

Therapeutic substances can penetrate the skin through two pathways: transepidermal and transappendageal. The first focuses on the passage of molecules through the stratum corneum, which consists of many layers of large, polyhedral, and unnucleated cells. Intracellular penetration can take place via the pathway involving corneocytes, which mainly allow the transport of substances with hydrophilic or polar properties, or through intercellular spaces that allow diffusion of lipophilic or non-polar substances through the lipid matrix. The second route, transappendageal, involves the passage of agents through the hair follicles and through the sweat glands [15]. Thus, topical application may result in the release of the drug into the skin and into the systemic circulation through percutaneous absorption including the passage of topically-applied molecules to the skin, percutaneous penetration during which the compounds move from the surface of the stratum corneum through the skin to the systemic circulation, and the permeation of chemical compounds through the skin by diffusion or through the pores [16].

The dermal absorption process of various biologically active substances, both cosmetic and pharmaceutical, is a process that can be divided into three stages: penetration, permeation, and resorption. Research to assess the amount of substances absorbed through the skin is useful to obtain qualitative and quantitative information on the number of chemical compounds, including cosmetics ingredients and drugs, that can enter the relevant systemic compartments in the body. Conducting such analyses is very important as it prevents the administration of a dose of compound that could have a toxic effect. Commonly conducted skin penetration studies using isolated skin for this purpose are due to the fact that one of its elements, the stratum corneum, is the main barrier that protects the body against penetration of foreign substances through the skin [17]. In vivo analyses on humans seem to be the most appropriate method used to assess dermal or transdermal drug delivery systems in humans. However, these kinds of experiments are associated with many obstacles, mainly ethical and economic. Therefore, methods that use human skin ex vivo have become a commonly used alternative to this type of research. This skin is usually obtained from corpses or patients undergoing plastic surgery. Another alternative is to use animal skin ex vivo as well as many different models of artificial or reconstructed skin that can provide results correlating with the results of in vivo human studies [18].

Due to the high demand for the development of new hydrogel biomaterials with increasingly better mechanical and therapeutic properties, various polymers, both synthetic and natural, are sought. A noteworthy starting material for the production of hydrogels that can be used in cosmetology and dermatology are natural polymers which, in addition to playing an important physiological and biological role in the human body, can also serve as substrates for the design of hydrogels. Natural polymers are particularly valuable due to their high biocompatibility, non-toxicity, similarity of their physical properties to natural tissues, numerous sites for modification with reactive groups, biofunctionality, and biodegradability. A very important feature is their relatively low immunogenicity which allows their use in some biomedical applications. Although these biopolymers are not always able to form a gel on their own, efficient cross-linking methods have already been developed, such as various chemical modifications, covalent cross-linking, and the use of gelling agents. Various chemical and physical design strategies for hydrogels have been developed to obtain hydrogels with desired properties. Among the most commonly used are enzymatic and disulfide cross-linking, supramolecular assemblies of guest–host pairs, click chemistry reactions, and supramolecular assembly through inclusion complexing. Recently, the efforts of researchers have focused primarily on the development of hydrogel biomaterials that, depending on the needs, would be versatile platforms with static or smart properties and responsive to stimuli. Due to numerous skin conditions that both cosmetologists and dermatologists struggle with, hydrogel materials are widely tested for their application in these areas. The current use of various types of hydrogels, which are useful in the treatment of various dermatological diseases and beauty deficiencies, focuses primarily on the use of hydrogel matrices as carriers of both topical and systemic medicinal substances in tissue engineering, cell therapy, or regenerative medicine [19,20].

In recent years, there has been growing interest in hydrogels for dermatology and cosmetology. They can be used in the technology of highly controlled active substance release systems by obtaining hydrogel materials. Due to inappropriate pharmacokinetic and physicochemical properties, many active substances are limited in local dermatology therapy or cosmetology procedures. However, these parameters could be improved by changing the dosage forms, such as through application of hydrogels [21,22].

Hydrogels have many features that give them a significant advantage over other forms of preparations used in cosmetology and dermatology. These biomaterials, due to their high swelling potential, have a similar degree of flexibility to natural tissues and may undergo gel-sol phase changes in response to various types of stimuli, both physical and chemical. The release of therapeutic substances from hydrogel structures can be activated at any time by changes in temperature, local pH, physical stimuli, as well as by the presence of various types of enzymes. There are also many possibilities to manipulate both the pore size and the surface properties of hydrogels to ensure adequate and controlled kinetics of drug release as well as to obtain a hydrogel with the mechanical properties desired for the application [23].

Thanks to the possibility of using electro-sensitive hydrogels, it is possible to improve and develop treatment methods based on the use of electrotherapy, which is popular in both cosmetology and dermatology. The use of this type of smart hydrogels may contribute to the controlled release of the therapeutic substance at the target site on the patient’s skin by adjusting the permeability and size of micropores under the influence of electrical stimulation [24]. Hydrogels can also support photodynamic therapy, which is used in both cosmetology and dermatology, thanks to the ability to change the properties of these biomaterials after exposure to light at the appropriate wavelength, which ensures controlled drug delivery [25]. Hydrogels as porous structures give the opportunity to incorporate into their structures a wide range of different types of drugs, differing in size or charge [26]. The possibility of sustained release of therapeutic substances by hydrogels allows for delivering a high concentration of active pharmaceutical substance to the target site for a long period of time. The big advantage of these biomaterials is also the ability to store and protect biologically active substances against the adverse effects of the external environment [23]. Properly designed hydrogels also allow minimally invasive filling of free spaces in the human body and delivering medicinal substances there due to the fact that their structures are similar to the extracellular matrix of many tissues [27]. By filling the space after damaged tissues and providing appropriate bioactive molecules they can also contribute to restoring new tissue [23]. Thanks to the ability to create three-dimensional polymeric hydrogels that are able to provide chemical and mechanical signals, these biomaterials can be an appropriate environment for the proliferation and differentiation of cells, which creates the possibility of delivering them to different places to restore damaged tissues [28]. The big advantage of hydrogel matrices is also the possibility of immobilizing on their surface or incorporating various enzymes used in the therapy of skin diseases, while maintaining their active and functional structure [23]. What is more, the possibility of designing hydrogels with high bioavailability and no immune response is also a great advantage of these biomaterials. In addition, due to the fact that many skin diseases as well as cosmetological or dermatological procedures are accompanied by the formation of many different types of wounds, maintaining a moist wound healing environment thanks to the use of hydrogels is extremely helpful in obtaining the desired results of treatment [29]. The use of hydrogels can also contribute to skin regeneration and thus reduce the formation of abnormal scars due to its biomimetic nature, regulated mechanics, and ability to crosslink at the target site. This is particularly important in the process of wound healing, especially in the case of burn wounds, where the availability of autologous skin is significantly limited [30]. Thus, the multitude of advantages that the use of hydrogel structures in the treatment of various dermatological diseases brings, makes them excellent biomaterials for versatile use.

In this review, we aim to present some of the main current directions in developing methods for obtaining, and further application of, polymeric hydrogels intended for dermatology and cosmetology, including therapies of various types of disease. In the review we critically analyze English-language scientific and professional literature, excluding patents.

## 2. Hydrogel Materials for Biomedical Applications

As is commonly known, hydrogels are polymeric networks with a three-dimensional configuration capable of imbibing high amounts of water or biological fluids. Hydrogels are widely used as medical materials due to its ease in manufacturing and self-application. They are used in contact lens production, cartilage reconstruction and regeneration, artificial organs, wound dressings providing the humid environment beneficial for wound healing, as materials for tissue engineering purposes, in plastic and reconstructive surgery as soft tissue fillers, and as augmentation materials. Due to their unique properties, hydrogels can also be used for drug release systems (DDS) [21,22,31,32,33,34,35].

Some hydrogels are called “smart hydrogels”. These hydrogels react to various chemical, physical, and biological stimuli (e.g., redox reactions, pH, specific ions, solvents, temperature, light, pressure, radiation, an acoustic, magnetic or electrical field, molecular recognition events) [21,35].

Various combinations of natural, semi-synthetic, and synthetic polymers are made into hydrogel formulations to use their potential as biomaterials. There are numerous applications of hydrogels in the medical and pharmaceutical sectors. Hydrogels can be used as contact lenses, membranes for biosensors, materials for artificial hearts or artificial skin, and active substances or drug delivery systems. They can also be used as carriers of drugs that can interact with the mucosa lining in the gastrointestinal tract, colon, nose, vagina, and various tumor tissues due to their ability to prolong their residence time at the delivery location [21,22].

There are different classifications of hydrogels. The division of these materials include, for example, the nature of the side group, their mechanical and structural characteristics, the method of preparation, the physical structure of the networks, and the mechanisms controlling the active substance release [31,34].

The various preparation methods of biomedical hydrogels are known. The physical, chemical and radiation cross-linking, and grafting-polymerization methods are used. Cross-linked networks of natural biopolymers such as alginate, carboxymethyl cellulose, carrageenan, chitosan, and hyaluronan, or synthetic polymers such as poly(acrylic acid) (PAA),polyethylene glycol (PEG), poly(ethylene oxide) (PEO), polyethylene glycol methacrylate (PEGMA), polyethylene glycol dimethacrylate (PEGDMA) and polyethylene glycol diacrylate (PEGDA), poly(hydroxyethyl methacrylate) (polyHEMA), polyimides (PI), poly(lactic acid) (PLAc), polylacide (PLA), poly(lactic acid) (PLAc), poly(vinyl pyrollidone) (PVP), poly(vinyl alcohol) (PVA), and polyurethanes (PUs) have been reported [21,31,34].

## 3. The Use of Hydrogels in the Treatment of Skin Diseases

In recent decades, significant progress has been observed in the development of biomedical hydrogels. The first developed hydrogels were mainly static implants, after which dynamic scaffolds appeared that could react to various biological stimuli. Subsequently, hydrogels began to be seen as potential carriers of biologically active substances, which was confirmed in many scientific reports, until they became platforms enabling cell proliferation and differentiation [36]. To date, various types of hydrogels have been developed that may find potential use in skin care and treatment of skin conditions. One type of hydrogel valuable for dermatology and cosmetology is bioadhesive hydrogel, which, thanks to its long residence time at the application site, reduces the frequency of application of a given product to the skin surface. One example of such hydrogels is the formulation proposed by Parenete et al., which was created by combining a carbomer homopolymer type C with xanthan gum that is also able to release caffeine gradually, which can be useful in the treatment of cellulite [37]. Another example is self-adhesive hydrogel patches based on sodium polyacrylate and carboxymethyl cellulose, which contain the active substance Triclosan, which is a well-known compound used in acne therapy [38]. Peel-off hydrogel masks are also used in skin care, which are dedicated to various skin types, including patients with sensitive skin, thanks to their cooling and soothing effects [39]. Silk sericin embedded in nanocellulose or hydrogels based on carboxymethylcellulose can be used to produce these types of hydrogel masks [40,41]. Another example is microcapsule-embedded hydrogel patches designed among others by Huang et al. whose application increases the permeability of diclofenac sodium through the skin, which in combination with ultrasound may support its permeability and improve the effects of therapy of local soft tissue damage [42]. Dressings are very promising forms of hydrogels, thanks to which it is possible, among others, to maintain a moist wound environment and the possibility of including antimicrobials and various biological signaling molecules in their structures. These structures can be used to treat both minor and chronic wounds, which often coexist with skin conditions [43]. Chemically cross-linked hydrogels have also found dermatological applications, an example of which is a hyaluronic acid-based hydrogel designed by Monticelli et al. This group developed a hydrogel based on this polysaccharide cross-linked by polyethylene glycol diglycidyl ether, which shows resistance to hyaluronidase naturally present in the skin, thanks to which it could be used as a filler in aesthetic procedures [44].Effective dermal and transdermal drug delivery is also a very important step in the treatment of various skin conditions, so more and more research is emerging to develop forms of hydrogels that can deliver drugs to the target place. Cellulose-derivatives-based hydrogels as vehicles can be a good solution that can efficiently deliver active substances through the skin [45]. Much attention from researchers around the world is focused on hydrogel scaffolds, which can act as cell scaffolds used to regenerate damaged tissues. Collagen proved to be a promising polymer for this application, which showed satisfactory interaction and imitation of biological functions [46]. Due to the fact that initially the hydrogels used inside the body were pre-formed externally and implanted using surgical techniques which was a very invasive procedure, research has focused on the development of hydrogels that can occur in situ after delivery by standard needles. This resulted in injectable hydrogels that reduce invasiveness and allow the delivery of biologically active substances and the filling of various tissue defects [47]. An example of this type of hydrogel, which can be used in cosmetology and dermatological procedures, is injectable shape-memorizing three-dimensional hyaluronic acid cryogels; they have shown very good properties in both in vitro and in vivo tests, so they can be used in soft tissue reconstruction [48]. As shown above, there is a wide spectrum of different forms of hydrogels that can be selected depending on the needs.

**Figure 1:**
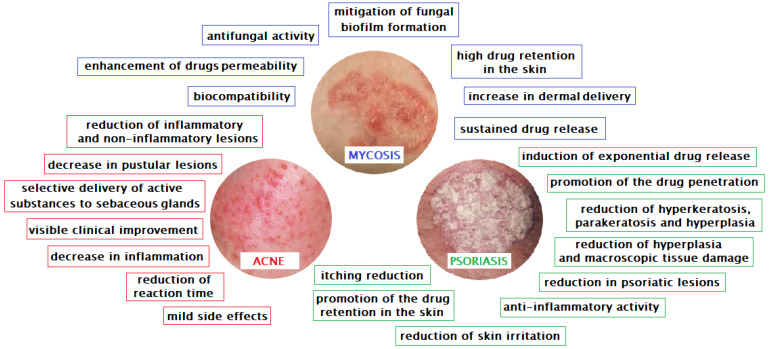
The effects of hydrogels in the treatment of selected skin diseases.

As mentioned before, one of the more interesting biomedical applications of hydrogels is used in dermatology and cosmetology. The use of hydrogels allows very effective treatment of many skin diseases and supports skin regeneration processes (Figure 1, Table 1).

### 3.1. Acne Vulgaris

Acne vulgaris is a complex, multifactorial, and chronic disease of the pilosebaceous unit, which occurs mainly in people under 18 years of age, but also affects many people between the ages of 20 and 40 [49]. Skin colonization by *Propionibacterium acnes*, ductal hyperkeratinization, the abnormal differentiation and desquamation of follicular keratinocytes, immunologic host reactions, inflammatory signaling and stimulation of sebaceous gland secretion by androgens are the main factors that play a very important role in the pathogenesis of this disease [49,50,51]. In addition, in appropriate eating habits and a poor diet or frequent stress can contribute to some extent to the development of this disease [51,52]. Drugs such as anabolic substances, steroids, neuropsychotherapeutic and cytostatic drugs also have an effect [53].

The method of treating acne is closely related to the severity of the disease. Topical therapy is mainly used to treat mild to moderate acne. In the treatment of acne lesions, retinoids and antimicrobials are primarily used [54]. The most popular drugs are benzoyl peroxide and preparations with antibiotics, whose main role is to inhibit existing acne lesions and prevent the formation of new ones [55]. The most popular forms of medicine for oily skin are gels, lotions, and solutions; for people with dry skin, lotions, creams, and ointments are more suitable. The main side effect that occurs when treating acne using these preparations is local irritation [56]. The use of retinoid therapy consists mainly of action on alveolar keratinocytes. This is to prevent excessive actinic keratosis and blockage and also to reduce the release of proinflammatory cytokines. Tretinoin, adapalene, and tazarotene are the most commonly used drugs in this group. Local antimicrobials, such as benzoyl peroxide, which kills bacteria by releasing oxygen into the follicle, are highly effective in treating this condition. Antibiotics, which are available in the form of preparations of various concentrations, are also very effective. As shown by the data, effective compounds from this group are erythromycin and clindamycin, which are topically administered and are well tolerated. However, it should be remembered that monotherapy with topical antibiotics should not be used routinely, because bacteria, including *P. acnes*, can become resistant very quickly [54]. To avoid resistance, a topical antibiotic with benzoyl peroxide is recommended. This treatment is used because the data indicate that combination therapy is more effective than using retinoids and antibiotics separately [57]. However, it is important to use these measures simultaneously only if they are compatible [54]. Many people struggling with acne, especially mild forms, use over-the-counter products. Among them, Proactiv, containing benzoyl peroxide, is very popular. Washing with 2% salicylic acid or using antibacterial soaps with benzoyl peroxide are also common methods [58]. In moderate to severe acne patients, topical medications are often insufficient, so systemic therapy is used. This includes oral antibiotic therapy, hormonal therapies, and the use of isotretinoin. Tetracyclines, erythromycin, minocycline, and doxycycline are very often used, which effectively reduce the number of inflammatory lesions [59]. Hormonal drugs are also used to treat acne. Studies show that estrogen-containing oral contraceptives and preparations that lower free testosterone levels give good results. On the other hand, progesterone-only contraceptives may increase acne lesions [55]. Ethinylestradiol and drospirenone, as well as ethinylestradiol with cyproterone acetate, have also shown quite good efficacy [60]. However, the use of combination therapy with topical agents or oral antibiotics brings better treatment results [54]. Isotretinoin is a very effective therapy, but not without side effects. It shows very good efficiency, because it can alter keratosis, reduce sebum secretion, inhibit *P. acnes* colonization, and has anti-inflammatory effects. This therapy should be used only in the case of very severe forms of the disease, because side effects include, among others, strong teratogenicity, hepatoxicity, hyperostosis, pancreatitis, erythema multiforme, epidermal necrolysis, or night blindness. It is also possible to use herbal therapies, such as tea tree oil or other oral herbal substances. Acne therapy may also include physical treatments, among which the popular methods are blackhead extraction, chemical peels, microdermabrasion, blue photodynamic therapy, and laser treatments for acne scars [54].

Due to the fact that one of the pathogenic factors responsible for the development of acne is skin colonization by various microorganisms, Lee et al. designed adhesive hydrogel patches for acne treatment containing the commonly used antibacterial drug Triclosan (TS). The developed hydrogel was based on sodium polyacrylate and carboxymethyl cellulose. To ensure greater penetration and accumulation of this drug in the skin, this group also incorporated Transcutol CG (TC) into the structure of the developed hydrogel. This compound as a penetration enhancer has been incorporated into the patch formulation. In studies focused on assessing the antibacterial properties of designed hydrogels against *P. acnes*, a bacterium closely related to the development of acne, they observed areas of growth inhibition on the plaque proportional to the content of antibacterial TC. The authors also drew attention to the fact that Triclosan as a hydrophobic compound will likelyeasily diffuse through the lipid layers of the skin, which increases its ability to penetrate and accumulate in the layers [38]. The legitimacy of using hydrogels in acne therapy has also been confirmed in clinical trials conducted by various groups of scientists [50,61,62]. In order to achieve the optimal effect of acne treatment, it is recommended to use a combination of atopic retinoid and an antibiotic, however until now these two classes of drugs have been used mainly separately [63]. The main obstacles are problems in the formulation of a preparation containing both these compounds, which significantly hinders the complete cure of this disease. A solution to this problem was proposed by Leyden et al. who developed hydrogels that can be carriers of both tretinoin (0.025%) and clindamycin (1%) in one preparation [61]. Clindamycin is a commonly used topical antibiotic used to reduce the proliferation of *P. acnes* and reduce inflammation, while tretinoin mainly normalizes and slows the desquamation process [64,65]. To confirm the applicability of the developed carriers in the treatment of acne, they conducted 12 weekly randomized, double-blind clinical studies on 2219 women and men. During these clinical trials, the subjects were divided into four groups and were treated with a clindamycin (1%) and tretinoin (0.025%) hydrogel, clindamycin (1%) hydrogel, tretinoin (0.025%) hydrogel, and hydrogel alone (vehicle). Research conducted by this group proves that the use of a combined hydrogel significantly reduces the number of inflammatory and non-inflammatory lesions compared to the other three types of hydrogels. They also observed a much shorter response time (time that resulted in a 50% reduction in the total lesion counts) and good tolerance of the developed hydrogel, which significantly improved the skin condition of people suffering from acne vulgaris. In these studies, no side effects of the applied hydrogel were seen in most patients, except for a few occurring at the application site such as dryness, burning, erythema, or irritation. In summary, the developed hydrogel that is a carrier of clindamycin and tretinoin may contribute to achieving better results of acne vulgaris treatment thanks to the possibility of reducing many causes of this disorder [61]. Standard treatments for acne vulgaris, including topical antimicrobial agents, retinoids, hormone therapy, and oral antibiotics, often face obstacles related to the inability to inhibit the proliferation of *P. acnes* strains, which often become resistant to antibiotics and prevent effective treatment of moderate to severe acne lesions [66]. In addition, the teratogenic effects of some of the retinoids used have contributed to the search for new acne treatments, including photodynamic therapy (PDT), thanks to which eradication of *P. acnes* and sebaceous glands has been observed through increased synthesis of porphyrin and free radicals [67,68].

The use of PDT in combination with hydrogels has been proposed by Fadel et al. as a treatment option for acne vulgaris. As part of randomized, controlled, and blinded studies, they used hydrogels containing liposomes with loaded methylene blue (MB) in patients with mild to moderate acne vulgaris [62]. Methylene blue due to its properties is perceived as a promising compound that can find application in photodynamic therapy of many disorders and serious diseases [69]. They showed that using this therapy significantly reduces the number of inflammatory and non-inflammatory acne lesions, by 83.3% and 63.6%, respectively. After 12 weeks, 90% of patients experienced a significant improvement in skin condition and a reduction in acne lesions and edema. Most patients undergoing this therapy reported no pain and no serious side effects except for slight discoloration in three patients. These authors also proved that the liposomal hydrogel is able to selectively deliver methylene blue to the sebaceous glands, thanks to which it can significantly contribute to the success of photodynamic therapy of acne vulgaris [62]. Research on the effectiveness of the use of PDT using intense pulsed light (IPL) in the treatment of this disease was also conducted by Moftah et al. The purpose of their research was to compare the effect of photodynamic therapy using liposomal methylene blue compared to the use of intensive pulsed light alone. Studies conducted on thirty-five patients with varying degrees of truncal acne vulgaris indicated that the use of MB significantly reduced inflammatory and non-inflammatory lesions. Although this therapy caused more pain and caused several side effects than the use of intensive pulsed light alone, as indicated by the Cardiff Acne Disability Index (CADI), patients were satisfied with the treatment as the skin condition improved [70]. Another group of scientists showed a positive effect on the effectiveness of acne therapy using a carboxymethylcellulose hydrogel with an incorporated natural compound trans-resveratrol. These studies demonstrated a much greater reduction of clinical lesions on the face after hydrogel with resveratrol application compared to the hydrogel alone. Patients also observed significant or even complete disappearance of macrocomedones, as well as a decrease in inflammation and pustules, which resulted in their satisfaction with the treatment [71].

Comparing conventional therapies used to treat acne and hydrogels as carriers of drugs used in its therapy, it can be concluded that the use of these biomaterials brings many benefits. A significant problem occurring during the treatment of this disease by traditional methods is local irritation, which, as shown in the studies cited above, can be minimized thanks to the use of hydrogel carriers of drugs. These carriers can also ensure greater penetration and accumulation of medicinal substances in the skin, as well as shorten the response time and ensure better drug tolerance. The use of hydrogels also allows the incorporation of retinoids and antibiotics into one matrix, which is often a problem with commonly used therapies, so that better treatment results can be obtained. As shown above, these biomaterials can also increase the effectiveness of photodynamic therapy and reduce the side effects of acne treatment.

### 3.2. Mycosis

Fungal infections, also called mycosis, are a very common problem affecting many people regardless of age, gender, or region. They also often coexist with other diseases such as asthma, acquired immunodeficiency syndrome (AIDS), cancer, organ transplantation, or corticosteroid therapy [72]. They mainly affect the skin and its appendages, because dermatophytes require keratin for growth, a protein that is found in the skin, nails, and hair [73]. Symptoms of a fungal infection of the skin include irritated, scaly, dry, red, and flaky skin that additionally itches and may be swollen. There are various fungal skin infections, among which are dermatophytosis, Candida and *Malassezia* infection, tinea capitis, fungal keratitis, and onychomycosis [74]. Although these diseases have been affecting people for a long time, optimal treatment has not yet been developed, although the recovery rates are quite good and usually range from 80–90% [75]. Commonly used antifungal systemic drugs often have serious side effects. When used topically, side effects are less common and less severe. The solution to various problems encountered in the treatment of fungal infections may be the use of controlled release systems for antifungal drugs, which, as studies show, can be achieved by incorporating drugs into hydrogel structures. By delivering the correct amount of drugs at the site of infection, these carriers can potentially achieve very high local drug concentrations without significant systemic distribution [76].

Currently, topical therapy, which is mainly used to treat local lesions, and oral therapy, which is used for more extensive fungal infections, are used to treat skin infections caused by dermatophytes. Although a wide range of antifungal agents have been developed so far, a huge problem interfering with complete recovery is the large irregularity in their intake and application, because many patients stop using antifungal substances after reducing symptoms, which very often causes remission of the disease. In dermatophyte infections, commonly used topical preparations with high efficacy include clotrimazole, tioconazole, econazole, isoconazole, miconazole, econazole, sulconazole, sertaconazole, and ketoconazole. Equally effective are substances that belong to allylamines, such as terbinafine, naphthifin, or butenafine. The available form of azole antifungal agents are usually creams, solutions, or sprays. The substances listed above rarely cause side effects and only in a few cases may allergic or irritating contact dermatitis occur. Commonly used oral antifungal agents with fairly good efficacy include terbinafine, itraconazole, fluconazole, ketoconazole, and griseofulvin. However, taking these drugs is often associated with various systemic side effects, including hepatitis. Due to the fact that in some cases a relapse is observed despite the initial good response to treatment, various treatment regimens are sought, which often involve a combination of oral and topical therapeutic substances [75]. In topical treatment of Candida skin infections, a number of antifungal agents are used, primarily azole drugs such as econazole, clotrimazole, ketoconazole, and miconazole, and polyene drugs such as nystatin, amphotericin B, and natamycin [77]. In contrast, fluconazole, itraconazole, voriconazole, and posaconazole are mainly used in oral therapy. Unfortunately, an important problem often extending therapy is primary resistance to these drugs found in some species such as *Candida albicans*, *Candida krusei*, *Candida dubliniensis*, *Candida glabrata*,or *Candida auris*. For the treatment of *Malassezia* infections, which are often the cause of variegated dandruff, mainly topical azole antifungal agents such as miconazole, clotrimazole, ketoconazole, sertaconazole, or allylamines such as terbinafine, naphthifin, and butenafine as well as ciclopirox are used. Oral administration of itraconazole or fluconazole is also effective [75]. Itraconazole is mainly orally used to treat folliculitis caused by *Malassezia* infection, since topical administration usually does not bring the expected results, probably due to poor penetration of the hair follicles. In seborrheic dermatitis, the topical application of ketoconazole, bifonazole and selenium sulfide has proved to be the most effective, and itraconazole can be used successfully in oral therapy [78]. On the other hand, the treatment of tinea capitis involves oral antifungal drugs such as terbinafine, itraconazole, griseofulvin, or fluconazole, which show different efficacy depending on the species causing the infection [75]. For example, in the case of *Trichophyton* infection, terbinafine shows good efficacy, whereas in the case of *Microsporum* infection, griseofulvin is more effective [79].

A major problem in the treatment of fungal infections of the nails is improper penetration of the nail plate by the drugs used. Therefore, antifungal therapy is often combined with the removal of an infected nail plate, by surgical excision or laser ablation, and also by the use of photodynamic therapy or iontophoresis which brings better results. Basically, the use of local therapy in the treatment of onychomycosis caused by dermatophytes is limited to cases where there is no involvement of the nail matrix and a significant thickening of the nail plate. Tioconazole and bifonazole, luliconazole, efinaconazole, ciclopirox, tavaborole, or terbinafine proved to be effective topical drugs, which are mainly used in the form of solutions, ointments, or nail varnishes. The effectiveness of these compounds are associated with the improvement of nail penetration due to reduced affinity for keratin and inhibition of the synthesis of enzymes and fungal proteins. Oral medications have proved to be more effective in treating this condition, among which terbinafine, itraconazole, fluconazole, and griseofulvin are quite effective. Unfortunately, the use of these drugs is associated with various gastrointestinal and nervous system side effects. Hepatotoxicity and skin rashes may also appear less frequently. Candida-induced nail dystrophy therapy is carried out using oral itraconazole, fluconazole or ketoconazole or chemically removed followed by topical antifungal therapy. In some cases, oral or topical antifungal agents are used in combination with topical corticosteroids. In cases of much less frequent nail infections caused by *Fusarium*, *Neoscytalidium*, or *Scopulariopsis*, a combination of oral and topical therapy is usually used. Methods of treatment using various medical devices, used alone or in combination with other topical or oral antifungal drugs, are also helpful in treating fungal infections. The most commonly used methods include ultraviolet radiation, photodynamic therapy, iontophoresis, and laser therapy. Surgical treatment is also used, which consists of mechanical or chemical debridement as well as surgical nail plate avulsion [75].

Due to the lack of an optimal treatment method for superficial fungal infections, which often also leads to many complications, Kumar et al. attempted to develop a topical hydrogel containing luliconazole nanocrystalline (LZL), which is a broad-spectrum local antifungal drug. As it is commonly known, fungal infections can affect various layers of the skin, so achieving a satisfactory result of treatment is conditioned by the delivery and retention of an appropriate dose of the drug both in the epidermis and dermis layers [80]. In recent years there has been a great interest in preparations based on various types of nanocarriers, including nanocrystals as potential carriers for various pharmacological substances. Studies show that nanocrystals have better properties than other nanoparticle systems. They show a high usable capacity of the drug, lower toxicity, and higher chemical stability [81]. Another advantage of these forms of the drug is that the preparation of the drug in the form of nanocrystals can significantly improve the bioavailability of the drug and its penetration through the skin layers, which is possible due to better solubility and prolonged retention at the site of infection [82]. Due to the low bioavailability of luliconazole as a result of its low water solubility, the study proposed the use of a hydroalcoholic hydrogel consisting of water and PEG. The use of PEG allowed for better drug dissolution and enhancement of penetration, while ethanol was used as a cosolvent which was supposed to facilitate the distribution of LZL in the hydrogel structure [83]. The results of the research received by this team indicate that the developed hydroalcoholic hydrogel can be used in the local delivery of LZL and has great potential compared to conventional formulations. In the conducted in vitro and ex vivo studies, they proved that the developed hydrogel carriers ensure very good drug trapping efficiency and improve its retention in the skin layers. In addition, this hydrogel has very good anti-fungal properties while being a safe carrier, causing only minimal irritation. Of course, prior to introducing the hydrogel to the market, further research is necessary to confirm the pharmacological activity, legitimacy of use, and safety of these carriers, while the results obtained by this team seem to be promising [80]. Sabale et al., as part of their research, developed a hydrogel preparation based on microemulsion using hydroxypropyl methylcellulose K100 as a gel matrix, which was responsible for stabilizing the system and increasing its viscosity [73]. The bifonazole asylum bond has been incorporated into the hydrogel structure, which is characterized by a wide spectrum of activity and effectiveness against yeast, dermatophytes, molds, and other fungi [84]. Due to the fact that the skin is a natural barrier that hinders local administration of the drug, this group proposed the use of microemulsions, which, according to scientific studies, are characterized by low skin irritation and high ability to load the drug. Microemulsions are also able to reduce the diffusion barrier and increase hydration of the epidermal stratum corneum. This is possible by dissolving lipids in the stratum corneum, which contributes significantly to increased drug penetration [85,86]. Therefore, they are seen as potential topical drug delivery systems that can further increase the bioavailability of poorly water-soluble active pharmaceutical ingredients [87]. The developed hydrogel was therefore aimed at improving the local delivery of bifonazole, which is an antifungal drug that is poorly soluble in water, by increasing its solubility and skin permeability. The obtained results indicate that the proposed microemulsion preparation increases the solubility and permeability of bifonazole through the skin, additionally showing very good stability. Moreover, this preparation showed very good antifungal activity and skin irritation comparable to commercially available bifonazole cream. Thus, the analyzed hydrogel has great potential as a carrier for sustained release of therapeutic substances, as well as those with poor solubility [88]. Research conducted by Zumbuehl et al. focused on developing a dextran-based amphogel with a loaded fungicidal compound. This agent was amphotericin B, which is widely used in clinical practice [89]. Amphotericin B is a broad spectrum polyene compound used to treat many types of fungal, mold, and protozoal infections. Its anti-fungal activity mainly focuses on binding to ergosterol and forming micelles in the fungal cell membrane, puncturing the cell membrane, activating lipid peroxidases, and inhibiting the membrane proton pump. The effect of this drug is strictly dependent on the dose used [90]. The analyses carried out by this group showed that the proposed hydrogels absorb amphoteric B into their structures and are able to kill C. albicans within 2 h of contact. Moreover, these materials can be reused for at least 53 days without losing their antifungal properties. Studies carried out using animal models have shown the biocompatibility of the hydrogel in vivo and showed that it is able to inhibit infections caused by C. albicans and the formation of fungal biofilm. Inhibition of biofilm formation is extremely important due to the fact that these biofilms can increase the resistance of microorganisms and make fungal infections resistant to the therapies used. The authors, in addition to the use of the developed hydrogels as independent antifungal systems, also indicated their potential use as an antifungal matrix, which can be used to coat various medical devices and implants [89]. The hydrogel containing amphotericin B was also developed by Hudson et al. The proposed material aimed at topical injectable antifungal treatment for direct administration. The solubility of amphotericin B in water was obtained by coupling with a dextran-aldehyde polymer. The analyses showed that this hydrogel has antifungal efficacy against C. albicans and provides antifungal activity in vitro for 11 days. Additionally, it has been demonstrated that exposure of C. albicans to hydrogel results in killing them for three weeks. The use of this hydrogel may help to ensure an adequate amount of the drug at the site of infection, thanks to which very high local concentrations of the drugs can be achieved, which will not undergo systemic degradation. The described in situ crosslinking hydrogels have many advantages, among which are ease of application, adapting to the shape of the space in which they are injected, long-lasting antifungal effects, and high biocompatibility. Thus, these hydrogels with incorporated amphotericin B may potentially be effective local antifungal therapy [91]. Other studies show that hydrogels can also become helpful tools supporting the action of photodynamic therapy of fungal infections, which is an effective way to treat infections caused by drug-resistant microorganisms [92]. This therapy involves the use of non-toxic photosensitizers in combination with light and oxygen in situ to create free radicals that are toxic to microorganisms [93]. Hydrogels formed by water-insoluble gelators such as bipolar lipids called bolalipids may prove useful here. Artificial single-chain bolalipids are capable of forming hydrogels due to the possibility of self-assembly, which is the result of hydrophobic interactions and stabilization by hydrogen bonds. Studies on the appropriateness of using such a solution were conducted by Goergen et al. who used two artificial bolalipids, PC-C32-PC and Me2PE-C32-Me2PE. The goal of this research was to design stable hydrogel preparations based on both bolalipids and to check their applicability as drug delivery systems with documented photosensitizing properties—methylene blue in the treatment of skin and mucous membrane infections. The release results obtained by this group showed that the developed bolalipid hydrogels showed sustained release of the drug. It has also been demonstrated that bolalipid methylene blue aerogels in combination with antimicrobial photodynamic therapy are able to inhibit growth and kill Saccharomyces cerevisiae. The possibility of using these matrices as a drug delivery system was also confirmed by biocompatibility studies performed on the surface of the cosmic membrane, which showed that both proposed bolalipid formulations showed excellent biocompatibility and high solidification capacity under body temperature conditions [92].

In summary, the use of hydrogels in the treatment of mycosis is justified because it allows to overcome or minimize many problems or side effects that are associated with the use of commonly used conventional treatments. The use of these biomaterials ensures the possibility of controlled release of antifungal drugs, which reduces the potential risk of achieving locally toxic concentrations of therapeutic substances as well as their high systemic concentration. As the above-mentioned studies show, they can also increase bioavailability, solubility, chemical stability, and penetration of drugs, which ensure the achievement of an appropriate therapeutic dose as well as their retention in individual layers of the skin. What is more, the hydrogels themselves can also exhibit antifungal activity, and those that cross-link in situ can adapt to the application site providing a long-lasting effect.

### 3.3. Psoriasis

Psoriasis is a chronic, multi-system inflammatory disease that primarily affects the skin and joints. Although the etiology of this disease is not yet fully described, research indicates that genetic and immunological background is of great importance [94]. Psoriasis can also occur as an isomorphic reaction in which the formation of psoriatic lesions is observed on previously normal skin that has been damaged by injury. The incidence of this disease varies from region to region. Greater incidence is observed in Europe and the United States, while in the case of the population living in Asia and Africa, the disease is observed less frequently [95]. The severity of psoriasis can vary throughout life and very often spontaneous flareups and remissions are observed [96]. Due to many unsightly changes on the skin of people suffering from this disease, it also contributes to increased levels of stress and avoiding contact with other people due to fear of rejection, which often greatly affects interpersonal relationships [97]. This disease is divided into various clinical types, the most common of which is chronic plaque psoriasis, which affects up to 90% of patients struggling with psoriasis. The characteristic changes that appear on the skin surface of people with this type of disease are symmetrical and erythematous plaques with an overlying silvery scale. Psoriatic lesions are observed primarily on the scalp, torso, upper and lower limbs, and buttocks, but can also appear on any other place on the human body. The resulting changes can be itchy or painful for patients. Psoriasis, being a chronic systemic disease, is also associated with the coexistence of many other diseases, such as cardiovascular diseases and malignant neoplasms [95]. Literature data indicate that it may also be associated with arthritis, depression, metabolic syndrome, and inflammatory bowel disease [98]. Accompanying diseases also include psoriatic arthritis (PsA), Crohn’s disease (CD), and uveitis [98,99,100]. Depending on the severity and type of disease, different treatments are used. In the case of mild to moderate severity of the disease, topical therapy is primarily used. It mainly uses corticosteroids, vitamin D3 analogues, and associated products. A slightly larger problem concerns patients suffering from more severe forms of this disease, who must be diagnosed by a dermatologist and treated using systemic therapy. Corticosteroids are common compounds used to treat this disease and are highly effective in mild psoriasis. Topical steroids that are currently used in various formulations, strengths, and combinations allow control of symptoms and disease management. Unfortunately, their long-term use may cause side effects in the form of mainly local skin lesions or tachyphylaxis [95]. Vitamin D3 analogues are also a very common therapeutic compound. One of them is calcipotriol, which is used as a first-line drug in the treatment of plaque psoriasis and moderately severe scalp psoriasis, which turns out to be as effective as most corticosteroids [101]. Other very effective analogues of vitamin D3 are beocalcidiol and paricalcitol. Therefore, they are commonly used alone or more often in combination therapy due to milder side effects including mainly mild irritant dermatitis and rarely hypercalcemia [102]. In order to achieve better treatment effects and alleviate side effects, combination therapy using both groups of drugs described above is also used. Studies show, for example, that the combination of calcipotriol and betamethasone dipropionate is more effective in the treatment of psoriasis than alone [103,104]. In addition to topical treatment, systemic therapy is also used, which mainly includes the use of phototherapy, acitretin, methotrexate, cyclosporin, or biologic therapy. Phototherapy is often used as first-line treatment, especially in moderate to severe psoriasis that does not respond to topical medications [105]. Acitretin is mainly used as adjunct therapy to other systemic drugs. It has been shown to increase efficacy and reduce the dose, minimizing the risk of side effects [106]. Less often it is used alone, as it is associated with undesirable effects including dry skin, gastrointestinal disorders, arthralgia, photosensitivity, and possible teratogenic effects [107]. The cytostatic and anti-inflammatory properties of Methotrexate have also contributed to its use in the treatment of moderately severe to severe psoriasis. It can also be used as an effective therapy for psoriatic arthritis. Although research indicates that it may have a positive effect on psoriasis treatment, it unfortunately shows hepatotoxicity and other adverse effects [108]. Ciclosporin is also a promising drug, which has a rapid onset of action and can be used to treat moderate to severe psoriasis. Although hepatotoxicity is not a major threat here, its use may be associated with other side effects such as nephrotoxicity, hypertension, elevated triglyceride levels, hyperkalemia, and even malignancies [109]. Another method of treatment, which has proven effective in the fight against psoriasis, in patients whose systemic therapy does not bring the expected results, is biological therapy which targets pathogenic T cells. This therapy mainly uses infliximab, ustekinumab, adalimumab, or etanercept [95]. As research shows, the use of these compounds induces in vitro apoptosis and selective reduction of the number of circulating cells, which improves the effects of treatment, without showing an increased risk of malignancy or infection [110,111]. Due to the fact that new treatments for psoriasis are constantly being sought that are highly effective and have the fewest side effects, alternative treatments using hydrogels are proposed below.

Limón et al. developed a bis-imidazolium based amphiphile hydrogel that can be used as a carrier for psoriasis therapies without side effects. They showed that this developed biomaterial provides protection of the drug against degradation and induces controlled release of such drugs as gemcitabine hydrochloride, methotrexate sodium salt, betamethasone 17-valerate, tacrolimus, and triamcinolone acetonide. Ex vivo penetration tests of human skin conducted by this group have demonstrated that synthesized supramolecular hydrogels with inbuilt drugs are able to effectively promote the penetration of these medicinal substances and their retention in the skin, which may increase their concentration in target tissues. In vivo experiments on mice also confirmed the possibility of using the developed hydrogels in the treatment of psoriasis, as they showed that these hydrogels contribute to a significant reduction of hypertrophy and tissue damage, which are very common changes in people suffering from psoriasis [103]. Tripathi and his group, on the other hand, conducted research on the potential use of Carbomer hydrogel bearing nanostructured lipid carriers that could carry Methotrexate (MTX), which in low concentrations acts as an anti-inflammatory immunosuppressant that can be used to treat inflammatory diseases such as psoriasis. Due to many side effects associated with the use of this drug administered in the form of oral or intramuscular preparations, these authors proposed the application of this drug in the form of preparations applied directly to the skin surface, which was to reduce the systemic response of the body. It is obvious that the topical application is very often associated with skin irritation, therefore the authors decided to entrapMTX in hydrogels with nanostructured lipid carrier (NLC) and solid-lipid nanoparticles. The obtained results indicated that the developed hydrogel carriers significantly reduced irritation in comparison to a conventional MTX loaded hydrogel. Due to the numerous problems associated with the penetration of various types of drugs through the skin, the use of solid lipid nanoparticles (SLNs) and NLCs as carriers of drugs, which show great similarity to skin lipids and quite high stability and the possibility of incorporating drugs of different molecular weight, may increase their deposition in the skin, which may result in prolonged release of therapeutic compounds in its layers [112]. Poorskin permeability of many medicinal substances used in psoriasis therapy significantly reduces their effectiveness at the target site of action. Researchers put a lot of effort into ensuring that the drug penetrates the skin sufficiently without compromising the skin barrier function. Thus, Baboota et al., in studies conducted to develop topical preparations for psoriasis therapy, assessed whether adding a compound with keratolytic activity, which is salicylic acid, and betamethasone dipropionate, which has immunosuppressive and anti-inflammatory effects, to a hydrogel based on microemulsions, will improve the delivery rate of corticosteroids. The hydrogel material developed by this group was characterized by good stability and a strong ability to penetrate medicinal compounds, which in the case of preparations used in the form of ointments, creams, or lotions was a huge problem. Importantly, this hydrogel is based on a microemulsion, whose advantages include low skin irritation and high drug release and loading capacity. Moreover, it did not contain additional chemical enhancers and solvents that can cause skin irritation and be harmful, especially after prolonged use [113]. Another group also conducted experiments on the development of nanostructured lipid carrier (NLC)-based hydrogel developed by the microemulsion technique. As an active substance incorporated into the hydrogel structure, they used mometasone furoate, which is a synthetic corticosteroid with anti-inflammatory properties mainly used topically to treat psoriasis. The development of alternative carriers for this substance, such as NLCs proposed by these authors, can contribute to increasing the effectiveness of psoriasis therapy, as the methods of transdermal delivery of this corticosteroid used so far have encountered numerous problems. The most important of them is the ineffective uptake of the drug due to the barrier, which is the stratum corneum and associated with numerous side effects such as swelling of the hair follicles or burning skin, and in some cases skin atrophy. NLCs, as improved forms of solid lipid nanoparticles (SLNs), ensure longer drug retention in the skin and allow for the elimination of the main disadvantages of SLNs which are limited capacity of drug loading and problems with regulation of its release, as well as drug expulsion during storage. According to literature data, they are also characterized by the ability to control and direct release of the drug, protect compounds embedded in their structures, as well as cause only slight irritation of the skin compared to other corticosteroid carriers such as creams, lotions, or emulsions. NLCs are potential carriers for improving drug retention at the target site and for reducing the risk of local and systemic side effects associated with topical corticosteroids. Thus, research by Kaur et al. indicates that the developed hydrogel carriers, such as mometasone furoate, can improve drug retention at the target site and reduce the risk of adverse effects, both local and systemic [114]. Available literature data also indicate that occlusive dressings may also have a beneficial effect on the treatment of psoriasis. They can be used both as monotherapy and also in combination therapy with topical medications. Although the mechanism of beneficial effects of these dressings is not fully understood, it is supposed to be associated with the restoration of the skin barrier, hydration of the epidermal layer, and reduction of mitotic activity of the epidermis in psoriatic plaques. Restoring the granular cell layer as well as maintaining an epidermal calcium gradient that is necessary for proper cell differentiation may also play a role. The advantage of using this type of dressing is the possibility of using a smaller dose of the drug, due to the increased bioavailability (e.g., topical corticosteroids). Therefore, Koo et al. conducted research to assess the legitimacy of the use and the feelings of patients with psoriasis treated with occlusive hydrogel dressings that would replace previous and current hydrocolloid dressings. This is important because initially used plastic occlusive wraps and adhesive tapes, and finally also hydrocolloids, were characterized by poor adhesion, irritation and skin injuries, tissue maceration or bacterial infections, and allergic reactions. Analyses carried out by this group showed that the proposed hydrogel patches show good efficacy, safety, and ease of use and are also positively perceived by patients, which makes them useful in topical psoriasis therapy [115]. Literature data indicate that hydrogels are not only applied as carriers of biologically active substances used in the treatment of psoriasis but may also be useful tools in understanding the etiology of this disease. Dutkiewicz et al. proposed the use of biocompatible hydrogel micropatch probes, which, in combination with mass spectrometry, would make it possible to study the composition of the skin metabolome of people suffering from psoriasis and could be helpful in resolving the pathophysiology of this disease. The development of this method is extremely important because the detection of skin metabolites is not currently frequently performed due to the lack of an efficient method, and the methods used so far using a cotton pad, wipe, skin-patch, or syringe are often associated with numerous inconveniences related to, among others, the necessity of performing chromatographic separation. Although there are several literature reports describing proteins excreted through psoriatic skin, little information is available on the psoriatic skin metabolome; hence the use of the proposed hydrogel micropatch probes can provide very valuable information [116].

Summarizing the literature data cited above, it can be concluded that the use of hydrogels in the treatment of psoriasis is most reasonable. In addition to reducing side effects resulting from the action of drugs used in its therapy, these biomaterials provide protection against degradation and allow highly controlled release. Moreover, the higher bioavailability of medicinal substances allows the use of a lower dose. Hydrogels can also effectively promote the penetration of many medicinal substances, often of different molecular weight, controlling their retention in individual skin layers and target tissues. The positive effect of treatment associated with the use of hydrogels may also be the effect of restoring the skin barrier, hydration of the epidermal layer, and reduction of the mitotic activity of the epidermis in psoriatic plaques.

**Table 1:** Application of hydrogels in the treatment of skin diseases.

Type of Skin Disorder	Type of Hydrogel	Agent	Animal Model/Cell Line/Microorganism	Mechanism of Action	References
Acne	adhesive hydrogel patches	Triclosan (TS)	in vitro: - female hairless mice skin (type SKH)	- antimicrobial effect of TS containing patches against *P. acnes* in the 0.01–0.3 wt.% concentration range in vitro - a significant increase in the amount of TS transported in hairless mouse skins	[38]
Acne vulgaris	clindamycin/tretinoin hydrogel	combination of clindamycin (1%) and tretinoin (0.025%)	clinical study: -randomized, double-blind multicenter clinical studies on male and female subjects	- greater reduction in the number of inflammatory and non-inflammatory lesions in the combined group compared to the other three treatment groups - significantly shorter response time (time to 50% reduction in the total lesion counts) - good tolerance of the fixed combination of clindamycin and tretinoin - significantly greater improvements in acne vulgaris	[61]
Acne vularis (facial)	liposomal methylene blue hydrogel	methylene blue	in vitro: - mice skin clinical study: - randomized, controlled and investigator blinded study on 13 patients	- significant reduction in the number of inflammatory and non-inflammatory acne lesions - moderate to significant improvement in acne in treated areas - no serious side effects - no edema - only slight temporary discoloration in three patients - selective delivery of BM(methylene blue) to sebaceous glands	[62]
Acne vulgaris (truncal)	liposomal methylene blue hydrogel	methylene blue	clinical study: - randomized and comparative study on 35 patients (21 males and 14 females) with varying degrees of acne vulgaris on the back	- significant reduction in the number of total, inflammatory and non-inflammatory lesions - some side effects such as pain, staining, pruritus, stinging, and flaking - greater efficiency LMB(Liposomal methylene blue)-intense pulsed light (IPL) than IPL alone	[70]
Acne vulgaris (facial)	carboxymethylcellulose-based hydrogel	resveratrol	clinical study: single-blind study on 20 patients (12 men and 8 women)	- reduction in the average area of microcomedones - decrease in inflammation and pustular lesions - no adverse effects - visible clinical improvement on the resveratrol-treated side of the face	[71]
Mycosis	hydroalcoholic hydrogel	luliconazole	in vitro: - *Candida albicans* (MTCC No. 183) ex vivo: - dorsal male albino Wistar rat skin	- enhancement in solubility - high skin retention - antifungal activity - increased dermal delivery	[80]
Mycosis	microemulsion-based hydrogel	bifonazole	in vitro: - *Candida albicans* ex vivo: - dorsal rat skin in vivo: - rabbits	- good stability of hydrogel over the period of three months - antifungal activity - sustained release of bifonazole - permeability enhancement of drug - improvement of solubility	[88]
Mycosis	dextran-based hydrogel (amphogel)	amphotericin B	in vitro: - *Candida albicans* in vivo: - male SV129 mice	- quick killing of fungi - no survival of fungi with amphogels - in vivo hydrogel biocompatibility - prevention of fungal infection - mitigation fungal biofilm formation	[89]
Mycosis	dextran-aldehyde hydrogel	amphotericin B	in vitro: - *Candida albicansSC5314* in vivo: - SV129 mice	- effectiveness in killing fungi on contact - long-lasting antifungal activity - biocompatibility	[91]
Mycosis	bolalipid hydrogel	methylene blue	in vitro: - *Saccharomyces cerevisiae*	- sustained drug release - antifungal activity - excellent biocompatibility - high solidification capacity under body temperature conditions	[92]
Psoriasis	supramolecular bis-imidazolium based amphiphile hydrogels	- gemcitabine hydrochloride - methotrexate sodium salt, - tacrolimus, - betamethasone 17-valerate - triamcinolone acetonide	ex vivo: - human excised skin in vivo: - male Swiss CD-1 mice with induced inflammation and hyperplasia	- suitable viscoelastic properties of the obtained hydrogel for topical drug delivery - significant influence of contained drugs on gel morphology at the microscopic level - protection of the drug by the hydrogel against degradation, - induction of exponential drug release - promotion of the drug penetration and retention in the skin - greater reduction of hyperplasia and macroscopic tissue damage *in vivo* compared to drug solutions	[103]
Psoriasis	carbomer hydrogel bearing nanostructured lipid carriers	methotrexate	in vitro: - Wistar stain albino rat skin in vivo: - rabbits	- effective incorporation of the drug into nanostructured lipid carrier (NLC) and solid lipid nanoparticle (SLN) hydrogel structures - reduction of drug release during storage - significant reduction in skin irritation in rabbits	[112]
Psoriasis	hydrogel transformed from microemulsion using Carbopol 934	- betamethasone dipropionate - salicylic acid	in vitro: - rat abdominal skin in vivo: - Wistar rats	- sustained and good anti-inflammatory activity - good stability, powerful permeation ability. and suitable viscosity of hydrogel - sustained drug release for the desired period of time - reduced dosing frequency	[113]
Psoriasis	nanostructured lipid carrier based topical hydrogel	- mometasone furoate	in vitro: - cellulose membrane ex vivo: - Wistar rats’ abdominal skin in vivo: - BALB/c female mice	- sustained release of mometasone furoate - negligible skin irritation - marked reduction in psoriatic lesions - lesser skin blackening - significant reduction of hyperkeratosis, parakeratosis, and hyperplasia	[114]
Psoriasis	hydrogel patch	-	clinical study: - men and women with plaque-type psoriasislesions	- intrinsic adhesion of the hydrogel - cooling and soothing properties - itching relief - safety and ease of use - no serious adverse events	[115]
Psoriasis	hydrogel micropatch	-	clinical study: - 100 psoriatic patients (75 men and 25 women) and 100 healthy volunteers	- the ability to analyze skin excretions using the hydrogel micropatch sampling approach combined with mass spectrometry - the possibility of using hydrogels as a non-invasive diagnostic tool for skin diseases - the ability to detect various skin biomarkers specific for psoriasis through the use of hydrogel micropatch	[116]

## 4. Wound Healing

**Figure 2:**
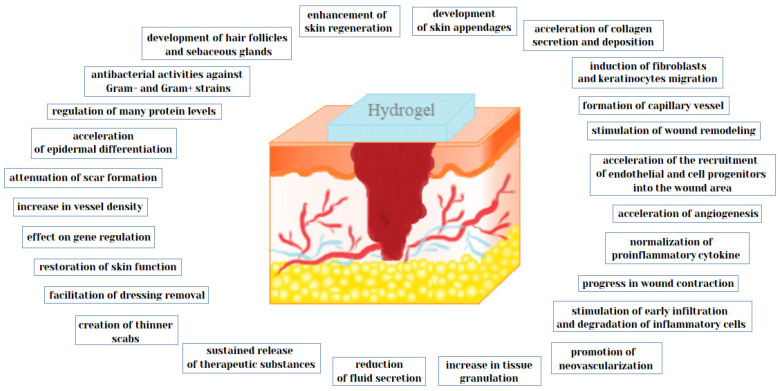
The effect of hydrogels on the wound healing process.

Non-surgical cosmetology and dermatological treatments are becoming more and more popular and are widely used by a wide range of people. Unfortunately, all these treatments lead to lesions of the skin to a greater or lesser extent. The big challenge is proper care and improvement of the wound healing process to minimize their visibility and prevent unsightly scars. It is important to use preparations that ensure optimal healing conditions after each procedure during which tissue breaks and skin damage occurs. A good strategy in this area is more likely to prevent complications and the appearance of unwanted extensive scars. Fortunately, modern dermatology and cosmetology by developing appropriate standards of care, including the use of hydrogel materials, and appropriate prevention, are able to effectively support the healing process of even the most problematic wounds. Many skin conditions that affect millions of people around the world are also closely related to the formation of various types of wounds, the treatment of which causes many problems. Due to the coexisting diseases and the action of many external factors, which can significantly delay and sometimes prevent the proper wound healing process, the demand for materials that can improve this process is constantly increasing. Thus, this paper also presents the possibilities of using various hydrogel matrices to improve the wound healing process occurring both in the case of various skin diseases and during treatments performed as part of therapy, which is extremely important in cosmetology and dermatology (Figure 2). Various natural and synthetic polymers or their composites are used in the technology of hydrogel active and inactive dressings (Table 2).

Proper wound care is an extremely important part of recovery, because it prevents the occurrence of unwanted infections and other complications, and also helps accelerate the healing process with less scarring. A wound is a disruption of epidermal integrity and the anatomical continuity of tissues or their damage under the influence of mechanical, thermal, or chemical trauma, which is accompanied by pain, bleeding, and opening of the edges of the wound. Wound healing is a dynamic process consisting of many chemical reactions involving numerous biologically active substances [117,118]. Not only the circulatory system but also the immune system and many other substances found in the human body are involved in the healing process. Wound healing is divided into three phases among which we distinguish inflammation, cell proliferation, and tissue remodeling [119]. There are many local and systemic factors that inhibit this process. Local factors include wound infection, necrotic tissue presence in the wound, drying the wound, excessive exudate, and insufficient blood supply to the wound area. Among the systemic factors, the most important are malnutrition, systemic infections, anemia, vitamin and calcium deficiencies, some medications, and various systemic diseases [117,118]. As proven in numerous works, moist healing ensures accelerated healing by maintaining the wound in an optimally moist environment. The use of hydrogels for this purpose helps to maintain an adequate wound moisture, which allows cell growth and migration. In a humid environment, keratinocytes can easily move around the surface of the wound, leading to its faster closure, and fibroblasts produce more collagen, which participates in the formation of the matrix for new tissue. The humid environment also provides hydration of the mucosal tissue and facilitates the autolytic cleansing process by retaining endogenous proteolytic enzymes in the wound. It also reduces the risk of wound infection by creating a hypoxic environment within the wound, which negatively affects the growth of potential pathogenic bacteria. Moist wound healing maintains growth factors in wound fluid that play an important role in initiating homeostatic cell response. This treatment also significantly reduces the pain associated with the wound and reduces the scars that result from injury [120]. The previously used dry dressings undoubtedly provide some protection, but due to their passivity, they are not able to respond to changing wound conditions or release drugs in a controlled or sustained way, which significantly contributes to accelerating the healing process. The concept of moist healing and the use of moisture-responsive materials such as films, foams, hydrocolloids, and hydrogels have greatly improved this process [120,121]. An extremely important advantage in the use of hydrogels in the treatment of wounds is also their limited adhesion, which means that they can be easily removed from the wound, without causing further injury to the treated tissue [122]. The multitude of available hydrogels ensures their usefulness in the treatment of many types of wounds. Among the natural polymers that have found application in wound care are polysaccharides such as alginates, chitosan, chitin, heparin, and chondroitin. Proteoglycans and proteins are also used, among which collagen, fibrin, gelatin, keratin, silk fibroin, and eggshell membrane are particularly important [123]. Hydrogel dressings based on natural polymers are excellent tools for treating wounds because of their unusual features, thanks to which they resemble soft physiological tissue. Among these properties, the most important are high molecular and oxygen permeability, low interfacial tension, good mechanical properties, and moisturizing abilities [124]. Due to the fact that polysaccharides have the ability to create biocompatible and biodegradable three-dimensional structures, they are considered by numerous groups of scientists as promising materials used to heal various types of wounds [125,126,127,128,129,130,131,132,133,134,135,136,137,138,139,140,141,142,143,144]. Muthuramalingam et al. examined the possibility of using a hydrogel prepared using immunomodulatory β-glucan. In addition to its good rheological properties, this hydrogel has demonstrated the ability to accelerate wound healing in both in vitro and in vivo analyses. In vitro studies have shown faster migration of keratinocytes and fibroblasts, and analyses carried out on a mouse model showed accelerated healing of skin wounds and better remodeling of newly formed skin tissue. The authors noted that the fact that β-glucan hydrogels increase wound healing may be associated with indirect macrophage activation or direct effects on keratinocytes and fibroblasts by inducing their proliferation and migration through specific receptors such as Dectin-1, CR3, or TLR. In addition, these authors pointed out the possibility of increasing wound healing and tissue regeneration by using these hydrogels as carriers for flavonoid compounds [126,145]. Research conducted by Sun et al. focused on the potential use of modified dextran hydrogels in the treatment of burn wounds by stimulating neovascularization and tissue infiltration in vivo, which largely determine the results of wound healing in deep burns. These authors showed that these hydrogels accelerate the recruitment of endothelial cells to the wound area, supporting neovascularization and skin regeneration with appendages [127]. Sun also attempted to develop bioabsorbable dextran-based hydrogels capable of modulating the late immune response by promoting the phenotype of anti-inflammatory M2 macrophages rather than M1 macrophages that induce immune responses and cause tissue destruction. His screening study showed that the immune-modulated dextran-isocyanatoethyl methacrylate-ethylamine (DexIEME) hydrogel is characterized by very good biocompatibility and promotion of the beneficial M2 phenotype. In studies carried out on murine and porcine models, he showed that the developed hydrogel promotes complete skin regeneration by creating a mature epithelial structure with skin appendages, which can lead to more efficient skin regeneration and reduced scar formation during deep wound healing [128]. Another group tested the possibility of using novel dextran hydrogel incorporating PEG-PLA (poly(lactide)-block-poly(ethylene glycol)) nanomicelles of curcumin in the treatment of full thickness wounds. Analyses showed that this hydrogel, in addition to its biocompatibility, also has the potential of sustained release of curcumin, which has proven wound healing acceleration properties [129,146]. In vivo studies using BALB/c mice have shown that the use of this hydrogel can significantly accelerate wound healing by promoting reepitalization, collagen deposition, angiogenesis, and fibroblast accumulation [129]. Considering the fact that protection against antibacterial infection is an extremely important aspect in wound healing, Lin et al. developed a poly(vinylalcohol)/dextran/chitosan hydrogel that showed dextran and chitosan concentration-dependent antimicrobial properties against both Gram-positive and Gram-negative bacteria. These studies also showed that the presence of dextran in the hydrogel structure provides a suitable environment for fibroblast growth due to positive charges and larger pore size of the hydrogel, which result in increased proliferation of these cells, which is important in wound healing [130]. Cellulose-based hydrogels are becoming more and more popular due to many beneficial properties such as hydrophilicity, high biodegradability, biocompatibility, good thermal/chemical stability, transparency, low synthesis cost, and lack of toxicity. Research is underway around the world on various strategies for developing efficient wound dressings that will have antibacterial properties through the use of a combination of antibiotics, biologically active substances, or antibacterial polymers [147,148]. Pinho et al. proposed the use of cyclodextrin/cellulose hydrogel with gallic acid to improve wound healing. Due to its antibacterial properties, this hydrogel can contribute to the prevention of wound infections, which significantly improves the healing process. Inhibition of bacterial growth at the wound site was associated with retained antimicrobial activity of gallic acid after introduction into the hydrogel, which results from its interaction with bacterial cell surfaces and reduced membrane integrity [125]. Fawal et al. developed hydrogels based on hydroxyethyl cellulose supplemented with tungsten oxide, which were characterized by anti-inflammatory and antibacterial properties. In addition, these hydrogels improved the safety of tungsten oxide relative to normal human cells in vitro, indicating that they can be an effective hydrogel dressing in wound healing [131]. Other studies focused on developing bacterial cellulose/acrylic acid hydrogels with the addition of human epidermal keratinocytes and skin fibroblasts, which were tested on burn wounds in athymic mice in vivo. The results showed that the addition of cells to the tested hydrogels significantly improves wound healing and increases collagen deposition in the tissue treated with the hydrogel. The use of a combination of keratinocytes with fibroblasts resulted from the fact that research shows that fibroblast cells can promote the growth and differentiation of keratinocytes by secretion of growth factor, while keratinocytes can provide signal molecules that stimulate fibroblast proliferation, which significantly contributes to improving wound healing [132]. Literature data indicate that self-healing hydrogels are a promising way to treat wounds. Studies show that they accelerate wound healing and can also be used as carriers of therapeutic agents, thus providing a local treatment effect. Research also demonstrated that cells embedded in self-healing hydrogels have the ability to proliferate and differentiate [145,146,147,148,149,150]. In research conducted by Cheng et al., long-term viability of novel chitosan, cellulose nanofiber self-healing hydrogels was studied. The results of conducted research showed that self-healing properties of hydrogels play a key role in tissue regeneration and this is probably related to the source of nutrients and oxygen in the tested hydrogel. These studies also showed that these hydrogels promote the regeneration of neurons in vitro and the regeneration of adult cerebellum injury in zebrafish in vivo [133]. Obara et al. demonstrated the possibility of using a photo-crosslink able chitosan hydrogel containing fibroblast-2 growth factor (FGF-2) in wound healing in db/db mice with healing disorders. The gradual release of FGF-2 during hydrogel biodegradation resulted in wound closure and restoration of normal tissue in vivo. The positive effect on wound healing was associated with the action of FGF-2, which according to literature reports stimulates the proliferation of fibroblasts and capillary endothelial cells, contributing to the formation of blood vessels and repairing wounds [134]. Tran et al. suggested the potential use of rutin-releasing chitosan hydrogels in the treatment of wounds, due to the promising results obtained both in vitro and in vivo in rats. The use of conjugated rutin with a chitosan hydrogel enables its prolonged release and overcomes its poor absorption during oral administration [135]. Yang et al. developed a series of hydrogels made from an aqueous solution of gelatin and carboxymethyl chitosan (CM-chitosan). The analyses carried out by this group indicated that gelatin/CM-chitosan hybrid hydrogels, due to their controlled biodegradation and in vitro cytocompatibility, can also be used as skin scaffolds and wound healing materials. The excellent cytological compatibility of the tested hydrogels can be associated with the high biocompatibility of gelatin and CM-chitosan as well as the ecological method of production of these biomaterials (radiation crosslinking) [138]. Hanzawa et al. developed a hydrogel consisting of a blend of alginate, chitin/chitosan, and fucoidan as a functional dressing providing a moist environment for rapid wound healing. The results of the performed analyses indicated that this hydrogel stimulates healing-impaired wounds repair caused by the use of mitomycin C in rats, while it does not have a significant impact on the healing of normal wounds [137]. Other authors demonstrated that the incorporation of chitosan hydrogel into honey can significantly enhance its antibacterial properties against many bacterial species and thus contribute to faster wound healing [138]. Chitin, despite its low solubility in most organic solvents, has also been used in the production of hydrogels, due to its useful biological properties, among which the most important are biocompatibility, biodegradability, hemostatic activity, and a positive effect on wound healing. Studies by Cho et al. showed that water-soluble chitin has a better effect on wound healing in rats compared to chitin and chitosan. This is probably due to its excellent biodegradability and hydrophilicity which may affect its better compatibility with wounded tissues and increase its activity in the process of wound healing [139]. Additionally, Straccia et al. demonstrated that alginate hydrogels coated with chitosan hydrochloride exhibit antimicrobial activity, lack of cytotoxicity, and are characterized by the possibility of prolonged release of biologically active substances, which can significantly contribute to increasing the efficiency of wound healing [140]. The purpose of the research carried out by Stubbe et al. was to obtain gelatin-alginate hydrogels with excellent mechanical properties, good biocompatibility, and the possibility of cell attachment. They showed that this goal can be achieved by selecting the appropriate ratio of gelatin to alginate, which results in obtaining a hydrogel with enormous absorption potential for exudate, maintaining a moist wound environment and increasing cell adsorption on the surface of the biomaterial. The authors decided to add alginate to this hydrogel because it is a biocompatible and non-immunogenic compound that can increase the swelling properties of the tested hydrogel, which contributes to the fact that it may find application in wound healing. Due to the lack of natural adhesion of cells to alginate caused by the absence of cell-interactive functionalities on the alginate back-bone, it is extremely important to choose the right proportions of ingredients to obtain the desired properties of the hydrogel [141]. Ying et al. developed a biocompatible and biodegradable hydrogel based on collagen I and hyaluronic acid characterized by a porous structure that contributed to the possibility of water retention, gas exchange, nutrition penetration, and cell maintenance, which in turn promoted spontaneous wound healing through the formation of blood vessels, reepithelialization, and production of collagen fibers [142]. Hyaluronic acid was also used for the production of hydrogels by Hong et al. The application of hyaluronic acid-based hydrogels in repairing skin defects in New Zealand rabbits resulted in promoting wound healing by changing gene activity, improving skin regeneration, and reducing scar formation [143]. Heparin is another polysaccharide used in the production of hydrogels. Heparin-based hydrogels consisting of thiolated heparin and diacrylatedPEG loaded with human epidermal growth factor (hEGF) have been shown to be effective in vivo for treatment of wounds in mice. Research conducted by Goh et al. demonstrated that these hydrogels are able to accelerate wound closure, promote capillary formation and reepitalization, and are characterized by prolonged release of hEGF in vitro [144]. Thus, polysaccharides are proving to be the right choice in the production of hydrogels used to improve the wound healing process. As the studies described above have shown, hydrogels based on these natural polymers not only provide excellent mechanical properties of hydrogels, but also the polysaccharide or its derivative itself can actively participate in the wound healing process.

Protein-based hydrogels also provide effective treatment for wounds of various origins. Particular attention should be paid to collagen, gelatin, and fibroin hydrogels due to their unusual properties. One of the most commonly used polymers in the production of hydrogels for wound healing is collagen, due to its biocompatibility, biodegradability, biological profile, and promising results both in vitro and in vivo [151]. Collagen also seems to be an excellent material due to its ability to recruit specific types of cells to the wound site, absorb exudates, maintain a moist wound environment, and stimulate the healing process by deactivating excessive matrix metalloprotease [151,152,153]. It should be noted, however, that collagen alone cannot cure infected tissues, due to the fact that it is a natural protein and bacteria can use it as a substrate [154,155,156]. Exploring the common problem of wound healing in patients with diabetes, Lei et al. conducted in vivo studies to examine the effect of collagen hydrogel on full thickness wound healing and promote capillary regeneration in diabetic rats. They showed that this hydrogel can promote wound repair, mainly by stimulating fibroblast proliferation, accelerating the synthesis of endogenous collagen in the wound area, and promoting angiogenesis, without any adverse effects. These authors also proved that the developed collagen hydrogel performs a similar role in wound healing as recombinant growth factors [157]. On the other hand, Elgharably and his research team conducted research on a modified collagen hydrogel dressing testing it on a pig model with chronic ischemic wounds. They demonstrated that this hydrogel increases the recruitment of macrophages to the wound in vivo. They also pointed out that the use of these hydrogel dressings upregulate the expression of Mrc-1 (reparative M2 macrophage marker) and induce the expression of anti-inflammatory interleukin (IL)-10and fibroblast growth factor (β-FGF) in vitro. In macrophages isolated from the wound, the authors also observed increased expression of M2 macrophage marker—CCR2. In addition, after 7 days an increase in regulation of transforming growth factor β, von Willebrand’s factor, vascular endothelial growth factor, and type I collagen expression was noted in the wounds, while after 21 days a greater number of proliferating endothelial cells was observed that formed mature vascular structures and increased wound blood supply. The use of the developed hydrogels resulted in an increased number of fibroblasts at the edge of the wound and an increased deposition of type I and III collagen, which resulted in tissue strengthening and prevented wound opening. Therefore, these studies indicate that these collagen hydrogel dressings have a positive effect on wound healing and induce angiogenesis and tissue remodeling [158]. In addition, the results of research conducted by Zheng et al. showed the possibility of using gelatin-based hydrogels blended with gellan as a valuable injectable wound dressing. Hydrogels developed by this group were characterized by the ability to promote cell adhesion and migration, and due to the content of tannin acid positively influenced the wound healing process due to their antimicrobial properties. The developed materials were characterized by the ability to control the release of antibacterial compound and high biocompatibility was confirmed by increased cell viability [159]. Other authors designed a gelatin hydrogel using the addition of alginate, which is a polysaccharide with numerous medical applications. To overcome the disadvantage of alginate, that is, its low solubility, Hoang Thi et al. used oxidized alginate. Proper reagents ratio in the developed gelatin/oxidized alginate-tyramine hydrogels was characterized by good swelling capabilities and reduced adhesion of these hydrogels, which facilitated their removal from the surface. In addition, the possibility of sustained release of H_2_O_2_ from the structure of these hydrogels affected their antibacterial properties. Moreover, the results of experiments carried out using three-dimensional (3D) cultures of human dermal fibroblasts indicated the biocompatibility of these matrices [160]. The possibility of achieving the correct wound healing process using fibrin gel was described by Murphy et al. These authors indicated that mesenchymal stem cells spheroids entrapped in fibrin gels may secrete pro-angiogenic and anti-inflammatory cytokines such as vascular endothelial growth factor (VEGF) and prostaglandin E2 (PGE2), and thus affect blood vessel formation and inflammatory processes. Interestingly, they proved that the secretion of these cytokines is closely related to the mechanical properties of these hydrogels [161]. Due to the good biocompatibility and bioactivity of the keratin protein derived from human hair, Kim et al. designed in situ cross-linked hydrogels based on this protein that have been shown to promote wound healing. In vivo studies showed accelerated reepitalization, tissue remodeling and repair, increased migration of keratinocytes, and upregulation of many genes, making these hydrogels a promising tool for wound healing [162]. Keratin was also used to fabricate hydrogels by other authors, while they used keratin derived from chicken feathers. The results obtained by this group indicate that these hydrogels positively influenced the wound healing process and were highly biocompatible, which was confirmed in experiments carried out on rats. In addition, they did not cause immune responses or systemic toxicity. These results suggest that not only human hair keratin-based hydrogels, but also feather keratin may find biomedical applications [163]. Silk fibroin is another protein that can be used to create hydrogels with potential use in wound healing, even in third-degree burn wounds. As Chouhan et al. have shown, in situ forming of injectable silk fibroin hydrogels can support the proliferation of primary human skin fibroblasts and the migration of keratinocytes in vitro. These biomaterials also initiate the transition of inflammation to the proliferation stage, by affecting the expression of TNF-α and CD163 genes and the deposition and remodeling of type I and III collagen fibers, which support tissue regeneration [164]. Silk fibroin has also been used to develop hydrogels that are carriers of human acidic fibroblast growth factor 1 (FGF1). In vivo studies of the effect of these hydrogels have shown accelerated dermis formation and differentiation of the epidermis into hair follicles and sebaceous glands. Increased collagen deposition was also observed after the use of these hydrogels, which contributed to an increase in scar healing rates. Furthermore, seeding cells on the surface of fibroin hydrogels increased their proliferation and migration [165]. The results cited in this article and summarized in Table 2 clearly indicate that both hydrogels based on carbohydrates and proteins have great potential and indicate that they can be used in the process of wound healing and tissue regeneration.

**Table 2:** Hydrogels for wound healing applications.

Type of Hydrogel	Analysis	Animal Model/Cell Line/Microorganism	Biological Activity	References
poly(vinyl alcohol) (PVA)/β-glucan (β-1,6-branched-β-1,3-glucan)	-determination of cellular morphology - assessment of cell viability - wound healing evaluation in vivo - assessment of skin regeneration (in vitro scratch wound healing assay) - evaluation of granulation tissue formation (hematoxylin and eosin staining) - epidermal thickness measurements - assessment of localization of cytokeratin proteins in the skin tissue - examination of protein expression (Western blotting	in vitro: - human dermal fibroblasts - human keratinocytes (HaCaT) in vivo: - mice	- wound healing acceleration - development of skin appendages in regenerated skin tissue - formation of capillary vessel - better granulation and reepithelialization - hierarchical arrangement of dermal layers - increase in expression level of transforming growth factor (TGF)-β3,cytokeratin 10 (K10), and cytokeratin 14 (K14) in the skin tissue - no detectable changes in cell morphology - migration and proliferation of keratinocytes and fibroblasts enhancement - skin regeneration - significantly faster wound closure - skin regeneration around the wound site - increase intranslational levels of K10, K14, and TGF-β3 proteins	[126]
dextran hydrogel	- evaluation of progress in wound healing - assessment of cell infiltration - assessment of macrophages and neutrophils accumulation - examination of aniogenic response - assessment of neovascularization - immunohistochemical analysis (Masson’s trichrome and alpha-smooth muscle actin staining) - assessment of blood flow surrounding the wound (laser Doppler) - analysis of the regenerated skin structure	in vivo: - mice - murine burn wound model	- promotion of remarkable neovascularization - promotion of dermal regeneration with complete skin appendages - facilitating early infiltration and degradation of inflammatory cells - promotion of the infiltration of angiogenic cells into healing wounds - mature epithelial structure with hair follicles and sebaceous glands development - acceleration of the recruitment of endothelial and cell progenitors into the wound area	[127]
self-crosslink able dextran-isocyanatoethyl methacrylate-ethylamine hydrogel (DexIEME	- evaluation of new skin regeneration in murine and porcine models - assessment of newly regenerated hair follicles - polarization of pro-inflammatory (M1)/anti-inflammatory (M2) macrophages - assessment of regenerative capacity in burn scars on mice - histological examination (hematoxylin and eosin staining) - assessment of regenerative capacity in deep full skin injury in pigs	in vitro: - human monocytic leukemia (THP-1)cell line in vivo: - murine and porcine models	- low pro-inflammatory response in murine and porcine models - regeneration of full skin structures with appendages on both pre-existing scars and acute wounds - promotion of complete skin regeneration with hair regrowth on preexisting scars - promotion of M2 macrophage phenotype - more mature wound - retention of the reticulated epithelial layer by new skin - more adipose tissue within the newly regenerated skin in both animal models - attenuation of scar formation	[128]
hybrid dextran hydrogel with incorporated curcumin encapsulated PEG-PLA(poly(lactide)-block-poly(ethylene glycol))	- evaluation of capacity to control curcumin release - histological study (hematoxylin and eosin staining) - collagen staining using Masson’s trichrome method - evaluation of neovascularization - assessment of expression of CD31 and vimentin in wound tissue	in vivo: - male BALB/C mice - murine full thickness wound model	- sustained release of curcumin from dextran hydrogel - significantly augment the reepithelialization of epidermis; increase in collagen deposition in the wound area - increase in fibroblast/mesenchymal cell density - acceleration of angiogenesis and fibroblast accumulation - increase tissue granulation - more mature epithelial structure with hair follicles and sebaceous glands - acceleration of the wound healing process	[129]
PVA/dextran/chitosan	- assessment of cell proliferation - determination of cytotoxicity (WST-1 assay) - evaluation of antimicrobial effect	in vitro: - mouse NIH-3T3 fibroblasts - *Escherichia coli* - *Staphylococcus aureus*	- antimicrobial ability to both Gram (+) and Gram (−) bacteria - no adverse effect on cell growth - dextran concentration-dependent stimulation of NIH-3T3 cell proliferation - improvement of cell adhesion to hydrogel with higher dextran concentration	[130]
cyclodextrin/cellulose hydrogel with gallic acid	- evaluation of antibacterial activity - assessment of cytotoxicity	in vitro: - 3T3 fibroblasts - *Staphylococcus epidermidis* - *Staphylococcusaureus* - *Klebsiellapneumoniae*	- sustained gallic acid release - significant reduction in the growth of all three bacteria - no cytotoxic effect on fibroblasts - no release of substances potentially toxic to fibroblasts	[125]
hydroxyethyl cellulose (HEC) supplemented hydrogel loaded with tungsten oxide (WO_3_)	- assessment of cytotoxicity of hydroxyethyl cellulose (HEC) with/without WO_3_ - determination of healing capacity - evaluation of anti-inflammatory activity(lipopolysaccharide-stimulated inflammation) - evaluation of antibacterial activity	in vitro: - human dermal fibroblast cells - white blood cells - *Shigella sp.* - *Salmonella sp.* - *Pseudomonas aeruginosa* - *Bacillus cereus* *-Staphylococcus aureus*	- antibacterial activities against pathogenic Gram-negative and Gram-positive strains - no morphological changes in the examined cells - improving tungsten oxide safety toward normal human cells (white blood cells and dermal fibroblast) - suppressed an abnormal immune response via normalization proinflammatory cytokine	[131]
bacterial cellulose/acrylic acid hydrogel containing keratinocytes and fibroblasts	- assessment of wound closure in vivo - histological analysis (hematoxylin-eosin and Masson’s trichrome staining) - determination of COL-1, CK-14, involucrin, and α-SMA expression in the tissue - assessment of regenerated skin ultrastructure (transmission electron microscopy)	in vitro: - human epidermal keratinocytes - human dermal fibroblasts in vivo: - male athymic mice(CrTac:NCr-Foxn1nu) -skin samples from six consenting patients	- acceleration on burn wound healing in vivo - confirmed by the presence of more mature keratinocytes - increase in involucrin expression - complete reepitheliation of the wound area by day 13 - decrease in cytokeratin 14 antibody and α-SMA expression - increased expression of collagen type I - more organized skin and collagen structures in the skin layer - a greater deposition of collagen in the mice - promotion of skin regeneration - the appearance of regenerated skin similar to normal skin	[132]
chitosan–cellulose nanofiber (CS–CNF) composite self-healing hydrogels	- investigation of cell proliferation rate by confocal microscopy and CCK-8 assay - evaluation of the oxygen metabolism and mitochondrial function of the cells - determination of nestin, glialfibrillary acidic protein (GFAP), CNPase, β-tubulin and microtubule-associated protein 2 (MAP2) gene expression for cells in chitosan–cellulose nanofiber (CS–CNF) hydrogels - assessment of nestin, GFAP, CNPase, β-tubulin and MAP2 protein expression of NSCs in CS–CNF hydrogels by immunostaining - evaluation of therapeutic function in vivo	in vitro: - neural stem cells (NSCs) from adult mouse brains in vivo: - adult wild-type zebrafish	- promotion of neural regeneration - the increase in the efficiency of mitochondrial electron transfer - strong relationship between oxygen metabolism and hydrogel self-healing properties - the increase in nestin, GFAP, CNPase, and MAP2 gene expression - lower level of β-tubulin (the early neural marker) - increase in the expression level of nestin, GFAP, CNPase, and MAP2 proteins - better regeneration in cerebellar injury of adult zebrafish	[133]
chitosan hydrogel containing fibroblast growth factor-2	- assessment of contraction and speed of wound closure in mice - evaluation of granulation tissue formation - assessment of capillary formation and epithelialization - histological examination (hematoxylin-eosin staining)	in vitro: - human umbilical vein endothelial cells (HUVECs) in vivo: - healing-impaired diabetic (db/db) C57BL/6female mice and their normal (db/+) littermates	- significant induction of wound contraction and accelerated wound closure in mice - accelerating wound closure by adding FGF-2 to the chitosan hydrogel in db/db mice, but not in db/+ mice - advanced granulation tissue and capillary formation - replacement of almost all necrotic tissues with new granulation tissue after injury on day 16 - significant epithelialization - stimulation of HUVEC growth, but loss of this ability after washing with phosphate-buffered saline (PBS) hydrogels for more than 3 days	[134]
rutin-conjugated chitosan-poly(ethylene glycol)-tyramine (RCPT) hydrogel	- evaluation of cell cytotoxicity - assessment of wound healing - evaluation of neoepithelium and granulation tissue formation - assessment of proteins formation	in vitro: - L929 mouse fibroblasts in vivo: - male Sprague-Dawley rats	- increase in fibroblasts proliferation at low concentration - very low cytotoxicity at high concentrations - increase in fibroblasts proliferation by releasing rutin - enhancement of wound healing - induction of better defined formation of neoepithelium and thicker granulation - more new blood vessels in wound - increase in the formation of extracellular proteins, primarily collagen	[135]
gelatin/CM-chitosan hydrogel	- assessment of hydrogel cytotoxicity - evaluation of the ability of NIH 3T3 cells to proliferate on or into the three-dimensional structure of hydrogel	in vitro: - L929 mouse fibroblasts - NIH/3T3 fibroblasts	- promotion of cell attachment on the hydrogel surface - acceleration of NIH 3T fibroblasts growth on the hydrogel - excellent cytocompatibility of the hydrogels	[136]
alginate/chitin/chitosan/fucoidan hydrogel	- assessment of hydrogel cytotoxicity and stimulatory effects on dermal fibroblast cells (DFCs) and dermal microvascular endothelial cells (DMVECs) - histological examination of removed skin and wound tissue samples (hematoxylin-eosin staining) - histological observations on repair of healing-impaired wounds	in vitro: - human dermal fibroblast cells (DFCs) - dermal microvascular endothelial cells (DMVECs) in vivo: - male Sprague-Dawley rats	- no cytotoxicity to DFCs and DMVECs - minor effect on healing of wounds not treated with mitomycin C - significant stimulation of repair of mitomycin C-treated healing-impaired wounds in rats - promotion of tissue granulation and capillary formation - positive effects on wound closure - progress in wound contraction and reepithelialization	[137]
chitosan hydrogel/honey	- assessment of antimicrobial activity - evaluation of granulation and fibrotic tissue formation - evaluation of inflammatory response - determination of reepithelialization - evaluation of wound shrinkage effects	in vitro: - *Staphylococcus aureus* - *Bacillus cereus* - *Escherichia coli* - *Pseudomonas aeruginosa* - *Candida albicans* in vivo: - male Wistar rats - rat full-thickness wound model	- significant decrease in the minimum inhibitory concentration against bacteria after the combination of a chitinase hydrogel with honey - significant improvement in the inflammatory index on days 3 and 7 - increase in granulation tissue formation - significantly higher fibrotic tissue formation index - increase in the level of reepithelialization	[138]
water-soluble chitin hydrogel	- histological examination of the wounded skins (hematoxylin and eosin staining, Masson’s trichrome staining) - determination of collagen-hydroxyproline in wounded skin - assessment of reepithelialization	in vivo: - animal model: rats	- acceleration of wound healing - significant reepithelialization - granulation tissue replacement in the wound by fibrosis - healing of hair follicles in rats - high skin tensile strength and the formation of the correct collagen fiber system	[139]
alginate hydrogels coated with chitosan	- assessment of cell morphology - determination of cytotoxicity - evaluation of antibacterial activity	in vitro: - human mesenchymal stromal cells (MSCs) - *Escherichia coli*	- unmodified cell morphology - no inhibition of MSC growth and proliferation - bacterial inactivation higher than 99% after 3 h - complete killing of bacteria after 24 h	[140]
gelatin-alginate hydrogel	- assessment of cytotoxicity (MTT assay) - determination of cell adhesion - live/dead staining (calcein acetoxymethyl–propidium iodide)	in vitro: - HFF-1 foreskin fibroblast cells	- time-dependent partial inhibition of cell viability - reduction of cell adhesion properties - presence of only living cells, no dead cells	[141]
collagen-hyaluronic acid hydrogel	- assessment of cytotoxicity (MTT assay) - evaluation of cells proliferation encapsulated into hydrogels at different time using confocal laser scanning microscopy - measurement of the cells’ attachment with scanning electron microscope - measurement of vascular endothelial growth factor secretion by human microvascular endothelial cells (HMECs) with enzyme-linked immunosorbent assay (vascular endothelial growth factor (VEGF)-ELISA) - assessment of antibacterial activity - measurement of biocompatibility and inflammatory in vivo - assessment of wound healing	in vitro: - human microvascular endothelial cells (HMECs) - fibroblasts (COS-7) - *Escherichia coli* - *Staphylococcus aureus* in vivo: - mice - mice full-thickness skin wound model	- good viabilities of HMECs and COS-7 cells - biocompatibility - excellent differential behaviors of cells in hydrogel - increase in the amount of vascular endothelial growth factor (VEGF) up to day 7 of culture - no foreign body reaction like immigration of giant cells into hydrogel - only few inflammatory infiltrations in the interface of hydrogel and tissue - gradual reduction in the number and infiltration of inflammatory cells - normal morphology with no signs of pathology for all organs, including heart, liver, spleen, lungs, and kidney - good adhesion of the hydrogel to the skin - killing about 55% of *E. coli* and 47% of *S. aureus* after incubating for 3 h - less colony-forming units of *E. coli* or *S. aureus* - disappearance of redness of wounds	[142]
hyaluronic acid-based hydrogels	- measurements of the white blood cell (WBC) level - histological staining with hematoxylin - immunochemistry analyses - determination of α-SMA, VEGF, and TGF-β1 gene expression (fluorescence-based quantitative PCR)	in vivo: - New Zealand white rabbits	- wound reduction - reduction of fluid secretion and thinner scab formation - faster recovery of the animals - increase in the level of white blood cells after surgery, reaching a maximum on day 7, followed by a decrease to a constant value - hair follicle renewal - increase in α-SMA expressions, which alleviated wound inflammation in the first few days after surgery and alleviated scar formation by reducing TGF-β1 levels. - faster self-healing compared to control, manifested by a faster increase in TGF-β1 level and higher VEGF levels after 7 days and lower after 14 days compared to controls - increase in α-SMA, TGF-β1, and VEGFmRNA levels from day 3, reaching the highest level on day 7 or 14,followed by a continuous decrease - increase in VEGF expression, which promotes skin regeneration through neovascularization	[143]
heparin-based hydrogel with loaded human epidermal growth factor (hEGF)	- evaluation of human epidermal growth factor (hEGF) release in vitro - histological examination (hematoxylin and eosin staining) - determination of the total amount of collagen in the regenerated skin(hydroxyproline assay) - immunochemistry staining with an anti-wide spectrum cytokine antibody by using LSAB+system-HRP kit	in vivo: - male BALB/c mice	- sustained release profile of hEGF in vitro - significantly accelerated closure of wounds - closing the epidermal layer and regenerating the extracellular matrix - denser epithelialization, blood vessel, hair follicles, and sebaceous gland formation - high similarity in the structure of the skin tissue of mice after injury after applying the hydrogel to normal skin tissue - accelerating wound closing and facilitating wound remodeling into normal skin tissue - increased proliferation and differentiation of keratinocytes - improvement of keratinocyte migration to the site of injury - hair follicles formation at the recovered tissue	[144]
collagen hydrogel	- assessment of wound morphology - determination of scarring shape and physical properties - histological examination (hematoxylin and eosin staining) - assessment of angiogenesis changes	in vivo: - Sprague-Dawley diabetic rat	- faster wound healing - increasing the speed and quality of full thickness wound healing in diabetic rats - significantly higher quantity of fibroblasts - increase in the number of new capillaries	[157]
collagen gel	- assessment of Mrc-1 (a reparative M2 macrophage marker) gene expression - assessment of anti-inflammatory cytokine interleukin (IL)-10 and fibroblast growth factor-basic (β-FGF)expression - immunohistochemical staining (with anti-vimentin and) DAPI)(4′,6-diamidino-2-phenylindole) - evaluation of mature collagen deposition - analyses of wound tissues	in vitro: - human THP-1 monocytes in vivo: - Yorkshire pigs - swine model of chronic ischemic wounds	- upregulation of Mrc-1 expression in vitro - induction of (IL)-10 and (β-FGF) expression in vitro - increased expression of CCR2 (M2 macrophage marker) - upregulation of transforming growth factor-β, vascular endothelial growth factor, von Willebrand’s factor, and collagen type I expression in ischemic wounds - increase in endothelial cells proliferation - significant increase in fibroblasts in ischemic wound-edge tissue - higher abundance of mature collagen	[158]
injectable tannic acid-loaded gelatin-based hydrogels blended with gellan gum	- evaluation of antibacterial activity using agar disc diffusion test - assessment of wound healing efficacy - histological examination (hematoxylin and eosin (H&E) and Masson’s trichrome staining) - measurements of epidermal thickness - assessment of skin tissue formation - evaluation of collagen deposition - examination of cell cytotoxicity and migration	in vitro: -murine L929 fibroblasts - *Escherichia coli* - *Staphylococcus aureus* - methicillin-resistant *Staphylococcus aureus* in vivo: - BALB/c mice	- long-term antibacterial efficacy - sustained release of tannic acid in vitro - significant wound reduction - no scars - skin fully covered with hair - 100% healing rate - increase in the thickness of the epidermis - effective granulation tissue formation - greater accumulation and collagen fiber content - effective skin regeneration and function restoration - increase in L929 cell viability	[159]
gelatin/oxidized alginate-tyramine hydrogels	- examination of tissue adhesive strength - assessment of residue H_2_O_2_ level - evaluation of antibacterial activity - measurements of cytotoxicity in vitro (WST-1 assay)	in vitro: - human dermal fibroblasts (hDFBs) - *Escherichia coli* - *Staphylococcus aureus* in vivo: - porcine skin	- high antibacterial ability due to the constant release of H_2_O_2_ - low adhesive strength of hydrogel allowing for the easy dressing removal - no cytotoxic effect on test cells	[160]
mesenchymal stem cells (MSCs) spheroid-containing fibrin hydrogels	- evaluation of cytokine bioactivity to stimulate endothelial cells and macrophages - assessment of anti-inflammatory potential - capacity of entrappedMSC spheroids to promote angiogenesis in a three-dimensional skin-like environment assessment	in vitro: - human bone marrow-derived MSCs - diabetic human microvascular cells (HMVECs) - raw 264.7 mouse macrophages in vivo: - human skin equivalent (HSE) model	- cytokine secretion with potential proangiogenic and anti-inflammatory effects - enhancing angiogenesis in the equivalent of human skin - increased endothelial cell penetration - stimulation of sprouts and greater invasion distance into the wound - dependence of VEGF and prostaglandin E2 (PGE2) secretion on the mechanical properties of hydrogels	[161]
human hair keratin-based hydrogel	- assessment of cell proliferation - immunocytochemical staining - evaluation of the cellular interaction of keratin with HaCaT cells - assessment of cell migration - measurements of gene expression	in vitro: - human keratinocyte cell line (HaCaT) in vivo: -male C57BL/6J mice	- stronger expression of vimentin and fibroblastic spindle shape in HaCaT cells - increasing the number of cells migrating into the space between confluent cell colonies - upregulation of the expressions of migration-related genes such as integrin αV, integrin α5, integrin β1, and integrin β6 - increase in mRNA expressions of integrin αV, integrin β5, integrin β6, fibronectin, Snail and vimentin - induction of molecular expressions of integrin β1 and vimentin - fully recovering the skin aspect without any signs of scab and skin contraction in animal test - reepithelialization of skin and the proliferation of dermal fibroblast with adnexa in dermis in vivo - effective wound remodeling with skin adnexa in vivo - high rate of the proliferation of dermal fibroblasts with skin adnexa and dense dermal fiber in vivo - full wound repair with skin appendages - regeneration of muscle tissue - increasing the number of hair follicles	[162]
feather keratin hydrogel	- evaluation of wound healing in vivo - assessment of cellular responses and vascularization (hematoxylin and eosin staining) - determination of collagen deposition in the wound skin (trichrome staining) - determination of the inflammatory cytokines IL-1β, IL-6, and TNF-α levels in the serum of rats (ELISA)	in vivo: - male Sprague-Dawley rats	- significant acceleration of wound healing - approximately 90% wound closure within 10 days - complete reepithelialization after 21 days - more hair follicles - more new capillaries around the inflammatory cells - maturation of the epidermis over the wound and restoration of new skin to normal after 28 days - acceleration of collagen deposition - thicker collagen fibers - no adverse systemic toxicity in rats after hydrogel implantation - no obvious organ damage and significant histopathological differences in the tissue organs in rats - no or minimal inflammatory response - no significant elevations in cytokines levels	[163]
injectable silk fibroin hydrogel	- assessment of cell migration - evaluation of wound closure - histological analysis - determination of collagen type I and III, TNF-α, CD68, CD163, and glyceraldehyde-3- phosphate- dehydrogenase (GAPDH) expression (qRT-PCR analysis) - determination of suprabasal keratin marker cytokeratin 10 (CK10), basal keratin marker cytokeratin 14 (CK14), and marker of terminally differentiated keratinocytes involucrin (INV) expression	in vitro: - HaCaT cells in vivo: - female Wistar albino rats - full thickness third-degree burn wounds	- complete wound closure after 21 days - two-fold accelerated healing rate in a regenerative manner - rapid development of granulation tissue - enhanced, early reepithelialization - mature epidermo-dermal regeneration - increase in HaCaT cells proliferation - formation of small blood vessels on day 7 - ten-fold increase in vessel density - significant upregulation and downregulation of collagen type I and III during different stages of wound healing - increase in collagen type I secretion from day 7 to 21 - higher secretion of collagen type III - visible expression of cytokines CK10 and CK14	[164]
heparinized silk fibroin hydrogels loading FGF1 (fibroblast growth factor 1	- assessment of platelet-derived growth factor (PDGF) and growth factor TGF-β expression (ELISA) - histological evaluation using hematoxylin and eosin and Masson’s trichrome staining - assessment of fibroblast L929 cells proliferation and migration	in vitro: - fibroblast L929 cells in vivo: - Sprague-Dawley rats - full-thickness excisional wounds	- increase in expression level of PDGF on day 7 - no statistically significant difference for expression of TGF-β1 - acceleration of dermis formation - acceleration of epidermal differentiation into hair follicles and sebaceous glands - improvement in small scars healing	[165]

## 5. Summary

The rapid progress in the technology of developing new hydrogel biomaterials, which can be used not only in biomedicine, but also in dermatology and cosmetology, is associated with countless combinations of various monomers/polymers from which hydrogels are obtained as well as medicinal substances that can be incorporated into their structures. As shown above, hydrogels, both natural and synthetic, have found application in the treatment of a wide spectrum of dermatological diseases, mainly due to the possibility of carrying therapeutic substances and providing a suitable environment for wound healing processes [166]. The most interesting and prospective seem to be the stimulus-responsive hydrogels, so-called ‘smart hydrogels’. Furthermore, stimulus-responsive hydrogels that act as sealants, adhesives, or dressings can provide proper wound healing that often coexists with skin conditions or is the result of dermatological or cosmetic intervention. They can become an important alternative to the currently used traditional sutures, staples, or dressings, the use of which does not often give the desired results [167]. In particular, the treatment of chronic skin wounds is a pressing problem that is almost reaching epidemic proportions, as it is estimated that by 2026 it may affect up to 60 million people worldwide [168]. Although there are currently many types of dressings designed to accelerate the healing process of wounds, those based on hydrogels are extremely popular, because as the research cited in this article shows, they provide a moist wound healing environment which accelerates the healing process and protects it against external factors, bringing very good treatment results [166]. The extraordinary advantage of hydrogels in the healing processes of many skin diseases is also their ability to provide specific substances to remove infections, such as antiseptics or antibiotics, and reduce inflammation by providing anti-inflammatory drugs or antioxidants [168]. Due to the use of encapsulation and cell culture on hydrogel matrices, these biomaterials have become particularly attractive for skin repair and regeneration [166].

Research involving the development of new hydrogel materials that can be used in cosmetology and dermatology is extremely important and desirable due to the fact that injuries and skin conditions affect millions of people around the world every year. This is a huge problem, because it is often associated with the formation of numerous scars and loss of skin appendages, and as it is commonly known, the use of even long clinical treatment does not always bring the expected results. The big challenge is therefore to achieve the effects of treatment and scar regeneration of the skin and full recovery of appendages, such as hair follicles and sebaceous glands. This paper shows that the use of different hydrogels for this purpose can provide a satisfactory treatment effect by repairing skin damage over the entire thickness. The unusual properties of hydrogels that can be used to treat dermatological diseases include primarily the ability to inhibit inflammatory processes, stimulate angiogenesis, improve the efficiency of wound healing, exhibit antimicrobial activity, prevent scar tissue formation by regulating cytokine expression, and recruit mesenchymal stem cells to injured places for the regeneration of skin appendages [166].

Due to the fact that research conducted over the past few decades has shown the versatile and extremely valuable properties of hydrogels, great progress has been made in their formulas and potential applications. Depending on the expected properties, it is very important to choose the proper hydrogel that meets the requirements. For this purpose, natural or synthetic hydrogels are used, which, as discussed in this paper, can be used in the treatment of skin diseases and also in the treatment of wounds, which very often are the result of skin diseases or a consequence of treatment methods. Very good properties, which are characterized by natural hydrogels contribute to the fact that they are very often used in the development of new matrices; this is because they are a kind of physiological hydrogels, which are characterized by very high biocompatibility and thus safety of use. It should be noted here, however, that due to their natural origin, the matrix composition may vary slightly between batches, and therefore their final microstructures and properties are sometimes difficult to control repeatedly and their mechanical properties are not quite satisfactory. Synthetic hydrogels have more flexibility in adjusting chemical composition or mechanical properties, while the conditions and methods of their polymerization should be strictly controlled in order to obtain a strictly defined structure or to give them hydrolysis or biodegradability properties after certain periods of time [169].

Obtaining a hydrogel with the desired properties also often requires the addition of a suitable compound that fulfills a specific function (e.g., therapeutic). However, due to the fact that the incorporation of a functional substance into the hydrogel structure is sometimes associated with a loss of its properties, it is important to choose the right matrix that will not lose the desired effect of the drug, and additionally protects it from mechanical, physical, or enzymatic degradation. For this purpose, for instance, the incorporation of a functional additive directly into the polymer as a comonomer is used [169]. Although the first hydrogels were developed 60 years ago, we have seen a dynamic increase in interest in these biomaterials in recent years. Their significant development, from the first generation hydrogels, which were obtained by cross-linking involving chemical modifications of the monomer or polymer with the initiator, through the second generation of stimulus-sensitive materials, thanks to which it was possible to properly polymerize the material, deliver the drug or create pores in situ, and finally the third generation hydrogels that are stereo complexed matrixes cross-linked by other physical interactions [169,170,171].

Treatment of skin diseases is a complex and lengthy process, due to their complex etiology, numerous side effects of applied therapies, and frequent problems in determining their primary cause. It should be noted that it is impossible to develop a hydrogel material that could find universal application in the treatment of all skin diseases, because many factors must be taken into account when designing such biomaterials. It is important to apply approaches that will be relevant in the therapy of a given disease entity and bring the best results. Unfortunately, to date, hydrogel materials are used primarily in the market of contact lenses, dressings, and hygiene products. However, their commercial use in tissue engineering and as drug carriers is still very limited, although many promising hydrogel matrices have been designed, tested, and even patented [169,172]. Perhaps this is due to the rather high cost of their production; nevertheless, constant development and overcoming various technical problems can minimize these costs, while obtaining valuable and effective biomaterials that will find wide application in the cosmetics and dermatological market. A wide spectrum of dermatological diseases, whose effective therapy is still a huge problem, is a big challenge for researchers working on the development of new hydrogel matrices; however, as shown in this article, there are numerous evidences that the use of hydrogels in their therapy offers many opportunities and perspectives.

The number of hydrogels which can be used in dermatology and cosmetology is still growing. However, there is a high demand for new hydrogel materials characterized by high biocompatibility, antibacterial properties, and ability to stimulate skin regeneration processes. Modern dermatology and cosmetology are awaiting innovative hydrogel materials characterized by highly controlled release of various active substances. For example, the use of these hydrogels potentially offers the opportunity of local administration of substances with antimicrobial properties, and thus the reduction of doses and a decrease in the systemic toxicity and side effects.

The treatment of skin diseases is very complex and challenging due to their pathophysiology. Hydrogel-based systems offer positively tremendous potential. In this review, current and potential applications of polymeric hydrogels (mainly hydrogel-based active substance release systems) in dermatology or cosmetology have been summarized. Hydrogel biomaterials should not only provide support for regeneration and skin repair processes but also achieve local highly controlled release of therapeutic substances.

Although considerable progress has been made in research and technology of biomedical hydrogels, many problems have yet to be resolved. The following points should be noted:

⮚ Results of biocompatibility analyses in vitro or even in vivo may not be sufficient due to the high complexity of the immune response and tissue repair processes in the human body.
⮚ Undergoing partial degradation, hydrogels may release toxic monomers, oligomers, nanoparticles, or molecules that may not be well excreted by the kidneys and accumulate in the body.
⮚ Protein adsorption on the surface of hydrogels, which occurs rapidly in vivo, can drive a biological response to skin-applied or implanted hydrogel materials.
⮚ Too fast and too slow release of immobilized active substances does not always lead to achieving the desired therapeutic effects.
⮚ It should be taken into account that in some cases hydrogel-based active substances can be difficult to store because of their low stability.
⮚ Antimicrobial strategies containing hydrogels to circumvent adverse effects or antibiotic resistance are under development. However, it should be proven that they are sufficient to eliminate chronic wound infections.
⮚ The use of hydrogels to treat extensive wounds results in an increase in the contact area and thus the material’s ability to interact with the body, which can increase immunogenicity.

The main challenges of biomedical hydrogels containing active substances involve:

⮚ Hydrogels incorporating active substances involved in the different healing stages and reparenting of skin have great potential. However, standardized protocols are needed in order to better understand their quality and to determine the amount and kinetics at which each active substance should be released.
⮚ Development of new hydrogels as drug carriers that can adsorb large amounts of drug molecules and provide better control over release kinetics.
⮚ Understanding the long-term effects of hydrogels in vivo to determine their potential clinical use.
⮚ Development of new hydrogel biomaterials that can increase the effectiveness of commonly used skin disease treatment procedures, such as photodynamic therapy.
⮚ Examining the effects of long-term use of hydrogel materials and a deeper understanding of the impact of their biodegradation products on the human body.
⮚ Development of efficient incorporation procedures and improvement of stability of various types of biologically active substances in hydrogels used in the treatment of skin diseases.
⮚ Reduction of side effects caused by drugs loaded in hydrogel structures and hydrogels themselves.
⮚ The possibility of mass production of biomedical hydrogels outside the scientific laboratory.
⮚ Simplifying the process of fabrication of biomedical hydrogels.
